# Differential Gene Expression in Macrophages From Human Atherosclerotic Plaques Shows Convergence on Pathways Implicated by Genome-Wide Association Study Risk Variants

**DOI:** 10.1161/ATVBAHA.118.311209

**Published:** 2018-09-06

**Authors:** Joshua T. Chai, Neil Ruparelia, Anuj Goel, Theodosios Kyriakou, Luca Biasiolli, Laurienne Edgar, Ashok Handa, Martin Farrall, Hugh Watkins, Robin P. Choudhury

**Affiliations:** 1 From the Division of Cardiovascular Medicine, Radcliffe Department of Medicine (J.T.C., N.R., A.G., T.K., L.B., L.E., M.F., H.W., R.P.C.), University of Oxford, United Kingdom.; 2 Nuffield Department of Surgical Sciences (A.H.), University of Oxford, United Kingdom.

**Keywords:** atherosclerosis, genome-wide association study, laser capture microdissection, lipids, macrophages, magnetic resonance imaging, transcriptome

## Abstract

**Objective—:**

Plaque macrophages are intricately involved in atherogenesis and plaque destabilization. We sought to identify functional pathways in human plaque macrophages that are differentially regulated in respect of (1) plaque stability and (2) lipid content. We hypothesized that differentially regulated macrophage gene sets would relate to genome-wide association study variants associated with risk of acute complications of atherosclerosis.

**Approach and Results—:**

Forty patients underwent carotid magnetic resonance imaging for lipid quantification before endarterectomy. Carotid plaque macrophages were procured by laser capture microdissection from (1) lipid core and (2) cap region, in 12 recently symptomatic and 12 asymptomatic carotid plaques. Applying gene set enrichment analysis, a number of gene sets were found to selectively upregulate in symptomatic plaque macrophages, which corresponded to 7 functional pathways: inflammation, lipid metabolism, hypoxic response, cell proliferation, apoptosis, antigen presentation, and cellular energetics. Predicted upstream regulators included *IL-1β*, *TNF-α*, and *NF-κB*. In vivo lipid quantification by magnetic resonance imaging correlated most strongly with the upregulation of genes of the *IFN*/*STAT1* pathways. Cross-interrogation of gene set enrichment analysis and meta-analysis gene set enrichment of variant associations showed lipid metabolism pathways, driven by genes coding for *APOE* and *ABCA1/G1* coincided with known risk-associated SNPs (single nucleotide polymorphisms) from genome-wide association studies.

**Conclusions—:**

Macrophages from recently symptomatic carotid plaques show differential regulation of functional gene pathways. There were additional quantitative relationships between plaque lipid content and key gene sets. The data show a plausible mechanism by which known genome-wide association study risk variants for atherosclerotic complications could be linked to (1) a relevant cellular process, in (2) the key cell type of atherosclerosis, in (3) a human disease-relevant setting.

## Abstract 2

Supplemental Digital Content is available in the text.

**Highlights**

- Macrophages laser captured from recently symptomatic human atherosclerotic plaques show differential gene regulation, which corresponded to 7 functional pathways, namely inflammation, lipid metabolism, hypoxic response, cell proliferation, apoptosis, antigen presentation, and cellular energetics.
- There were additional quantitative relationships between plaque lipid content measured by in vivo magnetic resonance imaging and key gene sets, particularly in the *IFN*/*STAT1* pathways.
- Cross-interrogation of gene set enrichment analysis and meta-analysis gene set enrichment of variant associations showed lipid metabolism pathways, driven by genes coding for *APOE* and *ABCA1/G1* coincided with known risk-associated SNPs (single nucleotide polymorphisms) from genome-wide association studies.
- The data show a plausible mechanism by which known genome-wide association studies risk variants for atherosclerotic complications could be linked to (1) a relevant cellular process, in (2) the key cell type of atherosclerosis, in (3) a human disease-relevant setting.

Macrophages accumulate in atherosclerotic plaque in response to lipid deposition, retention, and modification.^1^ They are involved in all stages of atherogenesis^2^ and are implicated in both plaque destabilization^3^ and lesion regression.^4^ Macrophages are notable for functional heterogeneity and their abilities to adapt in response to signaling cues and their microenvironment.^5^ They are also widely regarded as potential therapeutic targets.^6^

Until recently, views of macrophage plasticity have borrowed heavily from the T-lymphocyte T_H_1:T_H_2 paradigm in that T_H_1 cytokines, such as TNF-α (tumor necrosis factor-α), IL (interleukin)-1β, and IFN (interferon)-γ, induce polarization into M1 proinflammatory macrophages, whereas T_H_2 cytokines, such as IL-4 and IL-13, induce polarization into alternatively activated M2 (or reparative) macrophages.^7^ It is, however, increasingly recognized that additional cues may influence function and that characterizing plaque macrophages into discreet classes based on the expression of a few cell surface markers does not provide a sufficiently informative reflection of their in vivo functional roles^8^ and may obscure a wider phenotypic spectrum in both mice^7,9^ and humans.^10,11^ It is also recognized, largely from mouse studies, that one such cue is the accumulation of unesterified, free cholesterol, which promotes inflammatory pathways and cytokine secretion in macrophages through numerous mechanisms, including promotion of endoplasmic reticulum stress,^12,13^ NLRP3 (NOD-like receptor protein 3) inflammasome activation by cholesterol crystals,^14,15^ and accumulation of membrane cholesterol, which augments toll-like receptor activation that mediates inflammation.^16^

Studies of macrophage function in human atherosclerosis are less common, partly because of the challenges in isolating plaque macrophages from heterogeneous ex vivo tissue samples. Laser capture microdissection (LCM) allows highly enriched sampling of specific cell types from atherosclerotic plaque for RNA extraction and transcriptomic analyses.^17,18^ Furthermore, using LCM, cells can be precisely isolated from differing regions of the plaque, thereby providing insight into potential functional variation according to their sites of origin, for example, adjacent to (or within) the lipid-rich necrotic core (LRNC) vs the fibrous plaque cap. Here, we characterized the functional heterogeneity of macrophages within human carotid atherosclerotic plaques, aiming to identify pathways that are potentially differentially regulated in respect of (1) macrophage location in the plaque microenvironment, (2) the mode of clinical presentation of the plaque (stable vs unstable), and (3) an estimation of plaque lipid content with reference to in vivo T2 mapping using magnetic resonance imaging (MRI), before explantation.

Genome-wide association studies (GWAS) have provided a valuable, expanding collection of ≈161 loci associated with atherosclerotic vascular disease and its complications.^19–21^ However, GWAS identify only the genomic loci; to fully capitalize on the knowledge of these variants will require understanding of the modes of action, cell type(s) of expression, and biological effect of the associated variants.^22^ Accordingly, we tested whether genes within pathways that are differentially regulated in macrophages derived from stable vs unstable atherosclerotic plaques coincided with risk loci previously identified in human GWAS.

## Materials and Methods

Original data and material have been made available at the National Center for Biotechnology Information (NCBI) Gene Expression Omnibus (GEO) with GEO accession GSE118481. Gene Expression Matrix for the current study was also supplied in online-only Data Supplement.

### Study Population

Ethical approval was obtained from UK National Research Ethics Services. Study conduct conformed to the Declaration of Helsinki, and samples were stored in accordance with the UK Human Tissue Act (2004). Forty patients awaiting carotid endarterectomy at Oxford University Hospitals National Health Service Trust were recruited. Patients underwent MRI at the Oxford Acute Vascular Imaging Centre ≤24 hours before surgery. Carotid plaques were collected freshly at operation. The indications for surgery were either recently symptomatic carotid stenosis (median time from index event, 2 weeks) or asymptomatic carotid disease, with 50% to 99% stenosis according to NASCET (North American Symptomatic Carotid Endarterectomy Trial) or 70% to 99% according to ESCT (European Carotid Surgery Trial) criteria.^23,24^ Plaques were defined as symptomatic where they were deemed to have given rise to either a stroke or a transient ischemic attack as diagnosed clinically and supported, where available, by brain MRI/computed tomographic imaging. Asymptomatic carotid plaques were associated with no documented clinical symptoms but had constituted an indication for carotid endarterectomy, determined by the treating clinicians, based on %stenosis.

### Carotid MRI Protocol and Image Analysis

Patients underwent carotid MRI on a Verio 3T scanner (Siemens Healthcare, Erlangen, Germany) using published methods.^25^ Plaque lipid content was calculated using T2 mapping as described previously.^25,26^ T2 mapping allows voxel-by-voxel interrogation of lipid content, and the total lipid area calculated will include LRNC and smaller lipid pools along the vessel wall; although in established carotid atherosclerosis, the majority of plaque lipid measured will be within LRNC.

### Human Carotid Tissues and Quality Control

Explanted carotid plaques were immediately washed in ice-cold sterile PBS to remove blood, snap-frozen en bloc, and stored in −80°C for batch immunohistochemistry-guided LCM (Immuno-LCM; see the online-only Data Supplement for quality control).

### Immuno-LCM

LCM was performed using a PALM Microbeam LCM system (Carl Zeiss GmbH, Germany). A guide slide approach was adapted and modified from Feig et al.^4^ To discriminate cells based on immunophenotype and microanatomic locations, 1 frozen section (15 μm) was stained for Masson trichrome for plaque morphology, and 2 immediately adjacent frozen sections (15 μm) were stained with primary antibodies against CD (cluster of differentiation) 68 and smooth muscle α-actin in a rapid immunostaining protocol (online-only Data Supplement). LCM slides were then cut and individually stained with cresyl violet. Cell clusters of interest were identified based on guide slides by manual selection using PALM RoboSoftware (version 4.5; Carl Zeiss GmbH, Germany). For each cell type, a total combined area of interest between 3 and 5×10^6^ μm^2^ was procured from the subsequent (up to) 15 serial LCM sections, each 15 μm thick.

### RNA Extraction From Ex Vivo LCM Samples

Cells were laser captured onto Zeiss PALM AdhesiveCap (Carl Zeiss GmbH, Germany), and RNA was extracted using the RNEasy micro kit (Qiagen, Crawley, United Kingdom). The AdhesiveCap tubes were closed, inverted, and incubated for 30 minutes at room temperature. The lysates were then centrifuged at 12 000 rpm for 5 minutes. Samples were stored at −80°C for batch RNA extraction and purification. Total RNA was prepared using RNEasy micro spin columns (Qiagen, Crawley, United Kingdom), with on-column DNase I digestion to remove genomic DNA contamination. RNA concentrations and qualities were assessed using the Agilent RNA 6000 Pico LabChip on the Agilent Bioanalyzer 2100 (Agilent Technologies, Santa Clara, CA). Samples with RNA integrity number (RIN) <5 were considered of insufficient quality and excluded from downstream RNA amplification, biotinylation, and gene expression microarray analysis.

### RNA Amplification and Microarray

Human plaque macrophage RNA samples extracted by Immuno-LCM with RIN >5 were submitted to Cambridge Genomic Services (Department of Pathology, University of Cambridge, United Kingdom) for amplification and biotinylation using Ovation Pico WTA V2 kit (Nugen Technologies, Inc, San Carlos, CA) to minimize 3′ bias because of potential RNA degradation. Hybridization was then performed in random order on 2 Illumina Human HT12 v4.0 BeadChips (Illumina, San Diego, CA) to minimize batch/chip effects.

### Analysis of Gene Expression Profiles

Gene expression data quality control and preprocessing pipeline were performed using GenomeStudio (Illumina, San Diego, CA) with background correction and quantile normalization. GenePattern PreprocessDataset module was applied using default thresholding, filtering, and row normalization. Unsupervised principal component analysis was performed in GenePattern v2.0 (Broad Institute, Cambridge, MA). Differentially expressed genes were identified using T statistics with a fold change of >1.5 and a *P* value <0.05 for significance.

### Gene Set Enrichment and Leading Edge Analysis

Gene set enrichment analysis (GSEA) and leading edge analysis were performed using the GSEA v2.2.4 (Broad Institute, Cambridge, MA) with the Hallmark (containing 50 gene sets) C2 (containing 4738 gene sets) and C5 (containing 5917 gene sets) collections from the molecular signature database (http://www.broadinstitute.org/gsea/msigdb).^27^ Analysis was performed in January 2017, with a significant *P* value <0.05 and a false discovery rate (FDR) <0.25, according to the established criteria for GSEA.^28^

### Ingenuity Pathway Analysis

Ingenuity pathway analysis (IPA) was performed using IPA software (Qiagen, Silicon Valley, Redwood City, CA) as described in the online-only Data Supplement. IPA analysis was performed using differentially expressed genes with higher expression in symptomatic vs asymptomatic plaques (*P*<0.05; fold change, >1.4), as well as those with higher expression in plaques with large lipid core (≥25% lipid area on MRI T2 map) vs small lipid core (<25% lipid area; *P*<0.05; fold change, >1.5).

### Comparison With GWAS Datasets

Three hundred fourteen human SNPs (single nucleotide polymorphisms) with susceptibility association to coronary artery disease (derived from the interim UK Biobank report) and ischemic stroke^20,29^ were analyzed using Meta-Analysis Gene-Set Enrichment of Variant Associations (MAGENTA) v2.4 (Broad Institute, Cambridge, MA) with C2 and C5 gene sets from the molecular signature database (http://www.broadinstitute.org/gsea/msigdb).^27^ To determine whether there was convergence between significantly differentially expressed genes at a transcriptomic level from GSEA and loci identified by GWAS, the top 200 significantly enriched pathways from GSEA (containing 10 723 individual genes) were used as a custom superset to evaluate cross enrichment in MAGENTA using GWAS data; likewise, the top 30 significant enriched pathways from MAGENTA (containing 4854 individual genes) were used as a custom superset to evaluate cross enrichment in GSEA using transcriptomic data (online-only Data Supplement).

### Statistical Analysis

All statistical analyses were reported using mean and SEM, unless otherwise stated. The Gaussian distribution of all parameters was tested and confirmed. Differences in continuous variables between groups were compared using T statistics. Categorical variables were compared using χ^2^ tests. GSEA and MAGENTA analyses were performed as described previously.^28,30^ All statistical tests were 2 tailed, and a significant threshold was set as *P* <0.05. Statistical analysis was performed with GraphPad Prism, version 5.0 (GraphPad Software, Inc, San Diego, CA), and SPSS, version 21 (IBM Corporation, NY).

## Results

### Patient and Sample Characteristics

Thirty-two intact carotid plaques (16 symptomatic and 16 asymptomatic) were collected at the time of surgery. Four samples contained excessive calcification making intact cryosection impossible without decalcification (which would irreversibly degrade RNAs) and were, therefore, excluded from the study. Of the remaining 28 plaques, 15 were symptomatic, and 13 were asymptomatic. After further exclusion of samples containing suboptimal-quality RNA (RIN, <5.0), 12 symptomatic and 12 asymptomatic plaques were included in the final analysis. Table 1 summarizes patient characteristics. There were no significant differences between groups with regard to age, sex, cardiovascular risk factors, or medication at the time of carotid surgery. As reported previously, despite similar degrees of luminal stenosis (78.3±2.7% vs 82.9±2.7%; *P*>0.05), symptomatic plaques contained approximately double the proportion of plaque lipid (determined by in vivo MRI T2 mapping) compared with asymptomatic plaques (33.1±4.4% vs 15.2±3.5%; *P*<0.01). In addition, whereas some plaques contained macrophages exclusively in the juxta-core or near the fibrous cap region, other plaques contained both core and cap macrophages. The total pool of 24 plaques yielded 12 core samples (core macrophages) and 12 cap samples (cap macrophages). This was the basis of a 2-by-2 contingency comparison grid (symptomatic vs asymptomatic; core vs cap) each containing 12 samples. There was no systematic quantitative difference in the distribution of macrophages (core vs cap) between symptomatic and asymptomatic plaques in this cohort (see the online-only Data Supplement for comparison grid).

**Table 1.**
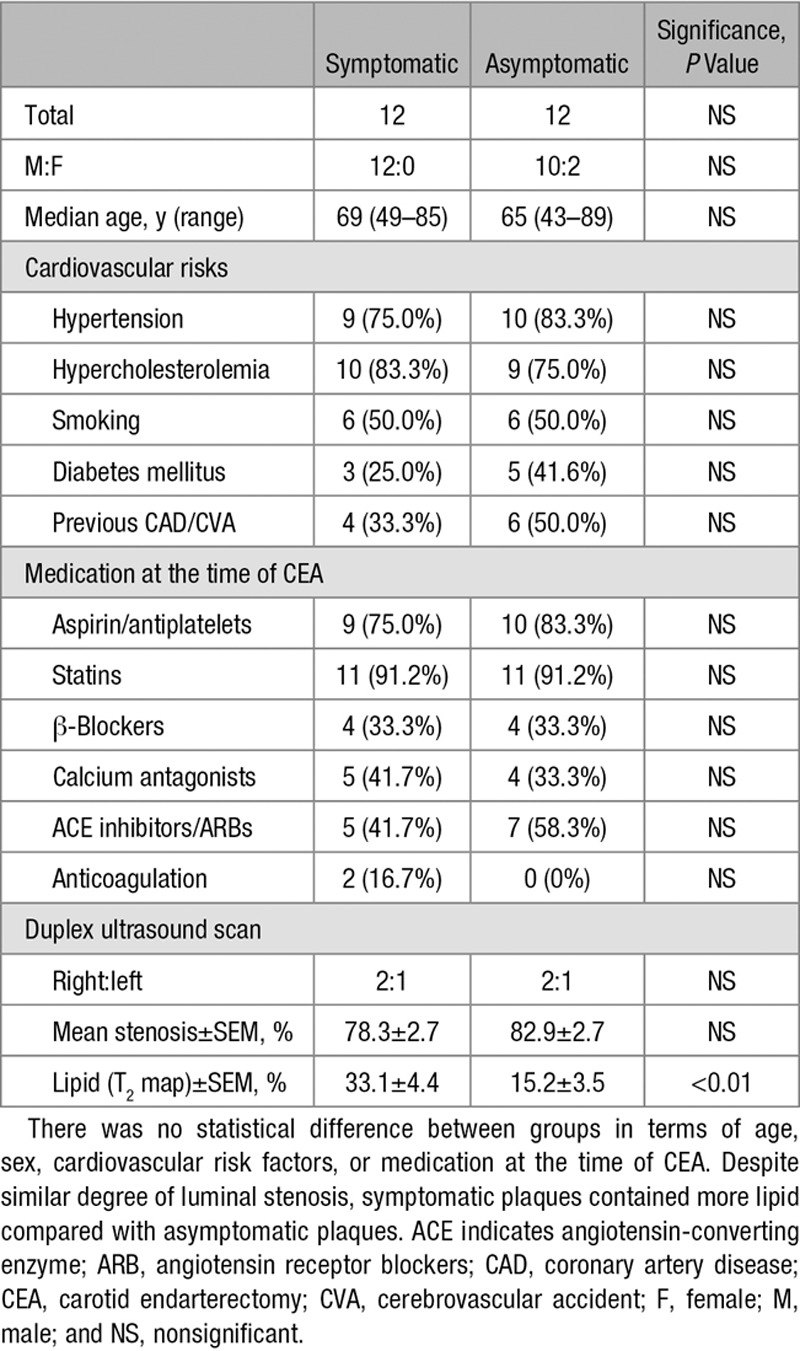
Patient Characteristics

### Immuno-LCM and Location-Selective Procurement of Macrophages

**Figure 1.**
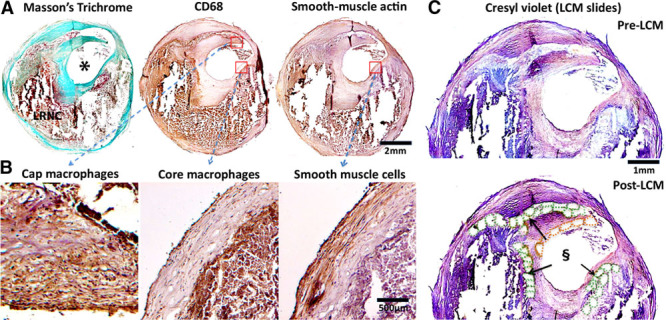
Immunohistochemistry-guided laser capture microdissection (LCM). Guide slides (**A**) provide anatomic and immunophenotypic information to locate plaque macrophages and neointimal smooth muscle cells. Closer examination (**B**) confirmed immunohistochemistry staining conformed with expected cell morphology of the selected cell types. **C**, Cresyl violet-stained LCM section on LCM slide (without coverslips, hence lack of color contrast) before (upper) and after (lower) LCM. Cutout elements on the post-LCM section correspond to laser-captured cell clusters. Green dotted elements, lipid core-associated macrophages; orange dotted elements, fibrous cap-associated macrophages. Reference to corresponding CD68 (cluster of differentiation) and trichrome staining from (**A** and **B**). Only cells with intact morphology were laser captured—necrotic material within lipid-rich necrotic core without intact cellular structure was not procured. *Lumen. §Cutout elements.

Cell procurement specificity with Immuno-LCM was first confirmed by isolating different cell types within the same tissue section (macrophages vs vascular smooth muscle cells; see the online-only Data Supplement; Figure in the online-only Data Supplement). Figure 1 shows an example of specific cell procurement. There was no statistical difference in RNA quality between symptomatic and asymptomatic plaques (RIN, 7.1±0.21 vs 6.9±0.21; *P*>0.05); likewise, there was no statistical difference in RNA quality between core and cap macrophages (RIN, 6.4±0.22 vs 7.0±0.22; *P*>0.05).

### Unsupervised Principal Component Analysis Exploration—Symptomatic Versus Asymptomatic Plaques

**Figure 2.**
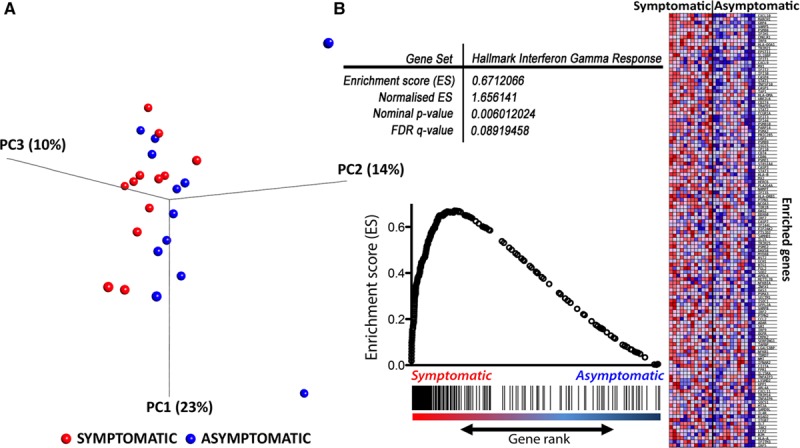
Principal component analysis and gene set enrichment analysis (GSEA). Principal component analysis (PCA) plot (**A**) showing the 3-dimensional representation of the first 3 principal components (PC1, PC2, and PC3 with percentages in parentheses referring to %variance by individual PC) with maximal separation of the data points. Each red dot represented a dataset from a symptomatic plaque, and each blue dot represented a dataset from an asymptomatic plaque. There was visual separation on the PCA plot except 3 outliers from the asymptomatic group (3 outliers, blue dots). **B**, An illustrative GSEA plot. Hallmark interferon gamma response is one of the most significantly enriched pathways in macrophages from symptomatic plaques with a *P* value of 0.006 and false discovery rate (FDR) of 0.09. Each individual gene in the ranked gene sets (vertical black line) was plotted against the Hallmark interferon gamma response reference gene set with a positive correlation with the gene set.

As a preliminary exploratory analysis, we first performed unsupervised principal component analysis, which showed a small group effect when comparing symptomatic and asymptomatic plaques (Figure 2A); however, there was no separation when comparing core and cap macrophages (online-only Data Supplement). We, therefore, further explored the dataset using GSEA on symptomatic vs asymptomatic comparison.

### Gene Set Enrichment Analysis

To fully capitalize the gene signatures of the whole transcriptome, especially to capture lower amplitude signals (eg, lower fold changes) from expression variation that would otherwise fall below the signal-to-noise significance cutoff (eg, including signal significance that might have been lost to adjustments for multiple statistical testing), we performed unbiased GSEA using whole macrophage transcriptomes. This approach de-emphasizes changes in individual genes in favor of understanding changes in specific pathways or functional clusters, which is considered more informative.^28,31^ At a conventional threshold of *P* <0.05 and FDR <0.25,^28^ 379 pathways were significantly enriched in macrophages from symptomatic plaques, of which 13 pathways were highly significantly enriched with *P* <0.01 and FDR <0.25. In contrast, only 1 pathway was significantly enriched in macrophages from asymptomatic plaques with thresholds of *P* <0.01 and FDR <0.25 (Figure 2B for illustrative GSEA; full list of enrichment pathways in Table I.II in the online-only Data Supplement). Pathways significantly enriched in macrophages from symptomatic plaques can be broadly categorized into upregulation of pathways involving (1) inflammation, (2) lipid metabolism, (3) hypoxic response, (4) cell proliferation, (5) apoptosis, (6) antigen presentation, and (7) cellular energetics, using categories derived from the Hallmark supersets collection, which represents well-defined biological states from overlapping gene sets (Table 2).

**Table 2.**
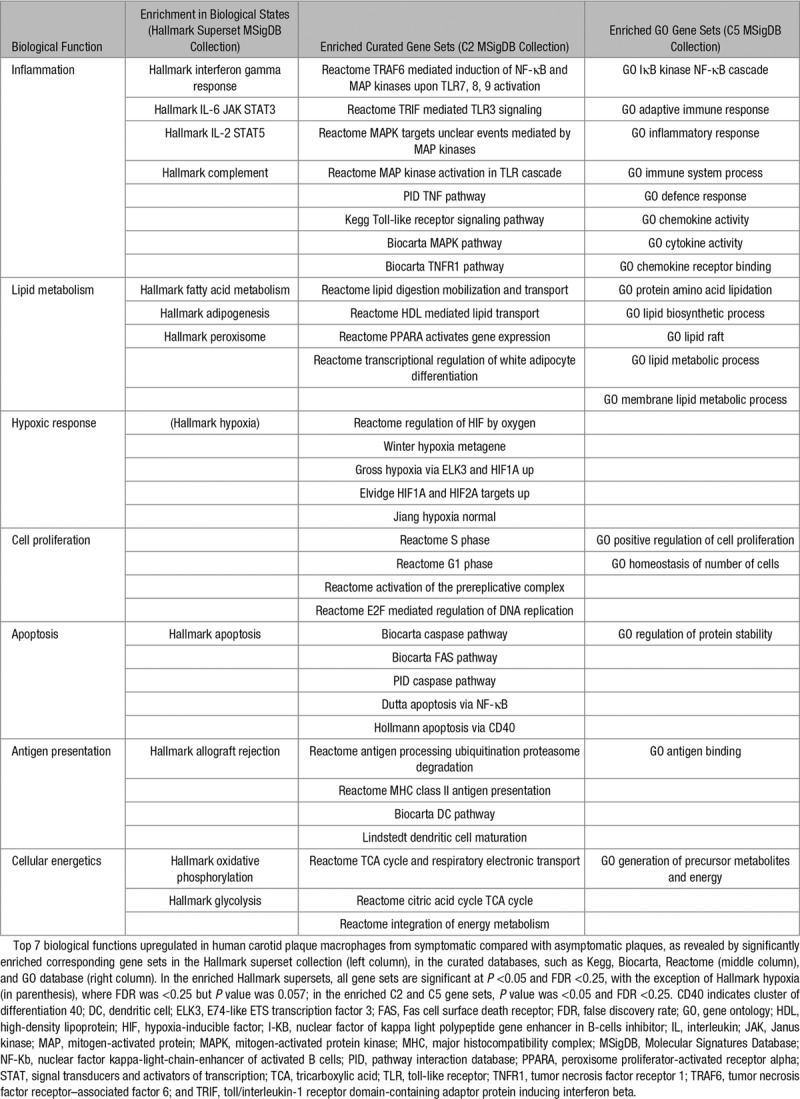
Upregulated Biological Functions in Macrophages From Symptomatic Human Carotid Plaques

### Leading Edge Analysis

**Figure 3.**
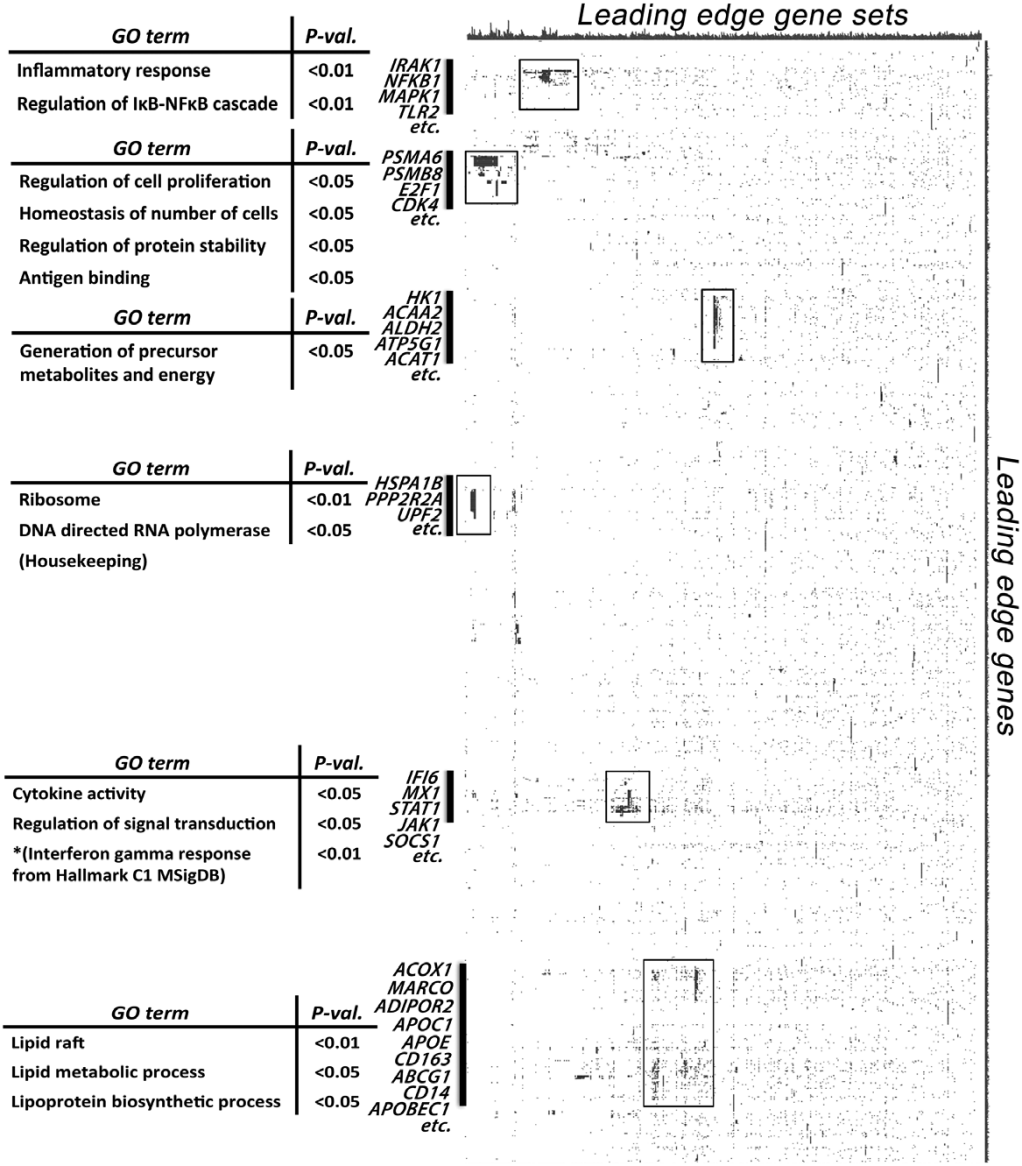
Leading edge analysis. Leading edge analysis identified major biological states upregulated in human macrophages from recently symptomatic carotid atherosclerotic plaques. These include inflammation, particularly cytokine response and interferon signaling; lipid metabolism; cell proliferation and apoptosis; antigen presentation; as well as cellular energetics. Each column represents a significantly enriched pathway (*P*<0.05; false discovery rate, <0.25), whereas each row represents a leading edge gene. GO indicates gene ontology.

**Figure 4.**
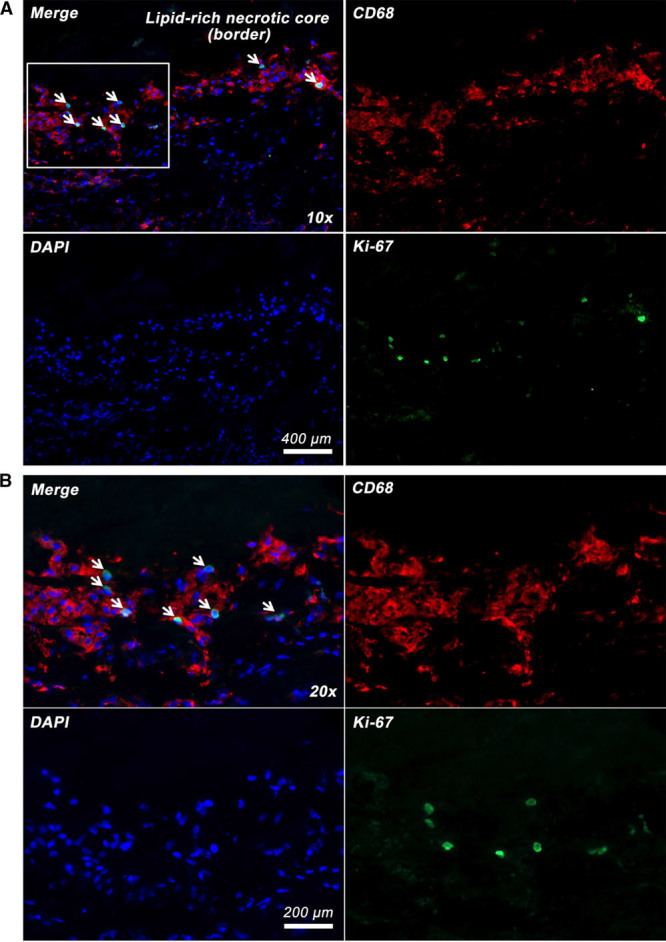
Coexpression of proliferative marker Ki-67 in human carotid plaque macrophages. Ki-67 (green) is coexpressed in the nuclei of some CD68 (cluster of differentiation)-positive plaque macrophages (red) at the border zone of lipid-rich necrotic core interfacing the overlying fibrous cap. Nuclear counterstaining with DAPI (4’,6-diamidino-2-phenylindole; blue). Boxed area in **A** shown as **B** at ×20 magnification.

We next examined the representation of genes present in the leading edges of the enriched gene sets in recently symptomatic plaques. The leading edge genes of an enriched gene set are those that contribute most significantly to the enrichment score and reflect the major drivers of enrichment. To gain further insights into the biological function revealed by these enrichments, we performed leading edge analysis of the significantly enriched gene sets (*P*<0.05; FDR, <0.25). We found multiple clusters of genes (vertical clustering) and gene sets (horizontal clustering) that broadly correspond to 6 of the 7 (except hypoxic response, which was not clustered) major biological states identified from GSEA described above (Figure 3). This suggests that these genes form part of transcriptional modules of coordinately regulated genes that are upregulated in human plaque macrophages from symptomatic plaques. Figure 4 shows an illustrative example of increased Ki-67 staining (for cell proliferation) at the border zone of LRNC interfacing the overlying fibrous cap.

### Enriched Pathways in Relation to Plaque Lipid Content by MRI

Because symptomatic plaques typically contain more lipid,^25^ we next sought to evaluate whether plaque lipid content was associated with differential gene set enrichment, which may influence the functional roles of plaque macrophages. First, the dataset from all plaques was divided into 2 groups: plaques with <25% lipid area (low lipid) and plaques with ≥25% lipid area (high lipid)—25% having been identified as a threshold associated with plaque instability in the literature,^32,33^ as well as from our previous study in a similar study cohort.^25^ GSEA using the C2 collection (with the highest number of enriched gene sets as shown above) revealed 72 pathways significantly enriched at *P* <0.05 and FDR <0.25 in plaques with high lipid content. The National Institutes of Health Pathway Interaction Database IFN-γ (interferon gamma) pathway was highly significantly enriched (*P*<0.01; FDR, <0.25). (Table II.I in the online-only Data Supplement) To further refine the enriched pathways, GSEA was repeated on core macrophages only because they are in direct contact with plaque lipid and are most likely to be affected by changes in lipid content—28 pathways were significantly enriched at *P* <0.01, of which 14 were involved in *TNF*/*IFN*/*STAT* (tumor necrosis factor/interferon/signal transducers and activators of transcription) signaling pathways.

**Figure 5.**
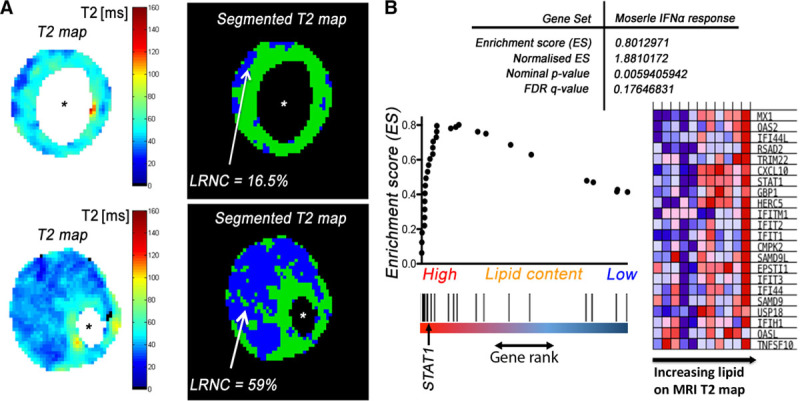
Gene set enrichment analysis vs T2 map lipid area. **A**, Representative raw (**left**) and segmented (**right**) T2 map derived from in vivo carotid magnetic resonance imaging (MRI). %Lipid-rich necrotic core (LRNC) calculated as segmented T2 map lipid area/total plaque cross-sectional area. **B**, An illustration of one of the most enriched pathways (Morserle IFN [interferon]-α response, C2 collection, Molecular Signatures Database) as plaque lipid content increases (*P*<0.01; false discovery rate [FDR], <0.25). *Lumen.

Next, instead of using a predefined threshold of 25% lipid area to divide the dataset, the dataset from core macrophages was reanalyzed using lipid area (%), measured directly by T2 mapping, as a continuous variable for GSEA. This approach quantitatively evaluated the degree of pathway enrichment/activation as the lipid content increases in the plaques, without bias to any predefined parameter. Five pathways were found to be significantly (*P*<0.05; FDR, <0.25) enriched as plaque lipid content increased, of which 3 were involved in IFN signaling; the most significantly enriched pathway being IFN-α response pathway (*P*<0.01; FDR, <0.25; Figure 5; also see Table II.II in the online-only Data Supplement). Leading edge analysis identified *STAT1* to be the top-ranking gene and contributed most to the enrichment signal.

**Figure 6.**
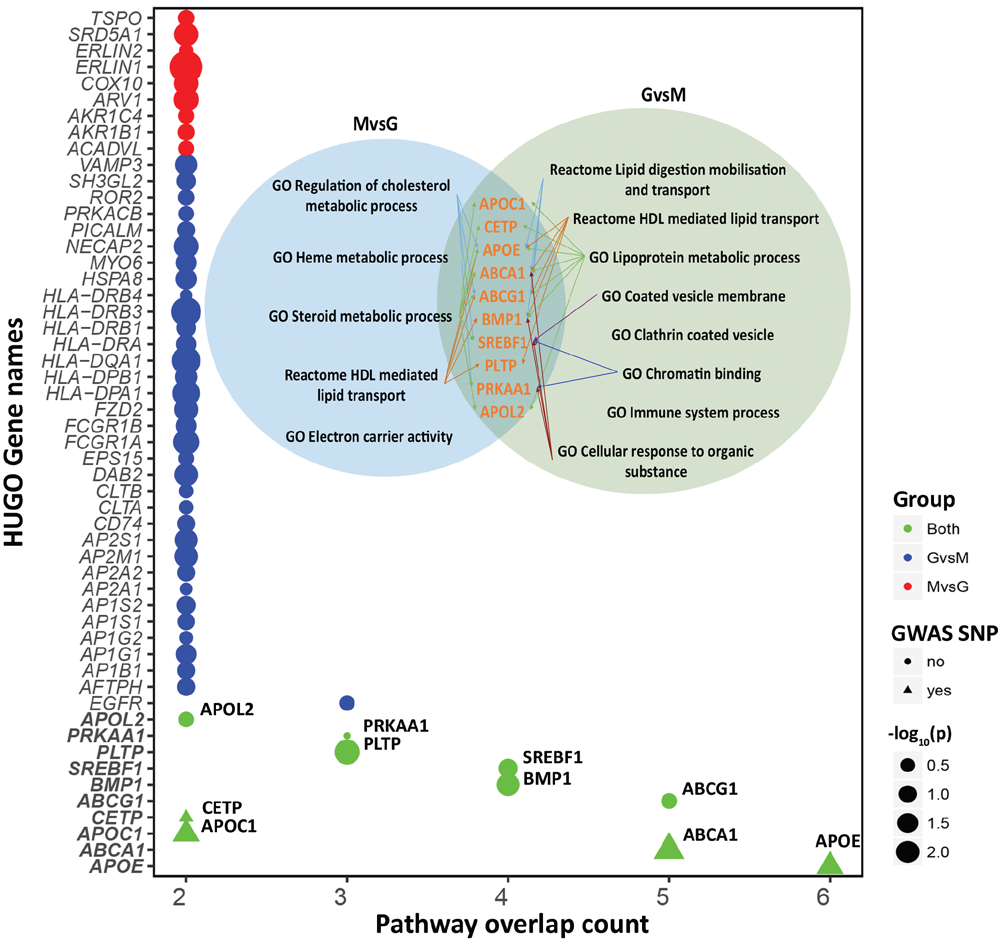
Overlap between GvsM and MvsG. Displaying the count of genes intersecting between the pathways found in GvsM and MvsG approaches (see Methods in the online-only Data Supplement). The genes are sorted by the absence or presence of a genome-wide association study SNP (single nucleotide polymorphism; circle/triangle). The size of the circle/triangle denotes the significance of differential expression in −log_10_(*P*); the colors show presence in GvsM, MvsG, or both groups. The genes that cross enriched between GvsM and MvsG are highlighted in the inset figures with their corresponding enriched pathways. ABCA1 indicates ATP-binding cassette transporter A1; ABCG1, ATP-binding cassette transporter G1; APOC1, apolipoprotein C1; APOE, apolipoprotein E; APOL2, apolipoprotein L2; BMP1, bone morphogenetic protein 1; CETP, cholesteryl ester transfer protein; GO, gene ontology; GvsM, GSEA vs MAGENTA; HDL, high-density lipoprotein; HUGO, Human Genome Organisation; MvsG, MAGENTA vs GSEA; PLTP, phospholipid transfer protein; PRKAA1, protein kinase AMP-activated catalytic subunit alpha-1; SNP, single nucleotide polymorphism; and SREBF1, sterol regulatory element-binding transcription factor 1.

### Exploration of Upstream Effectors and Downstream Functions Using IPA

To further explore the upstream effectors and downstream functional consequence of the LCM-transcriptomic microarray findings, and to corroborate cross-platform agreement and consistency of the bioinformatic analysis, we performed IPA on the differentially expressed genes (see Table III.I and III.IV in the online-only Data Supplement). In symptomatic plaques, IPA-predicted upstream regulators are in agreement with GSEA results, involved mainly in inflammation (eg, *IL-1β*, *IL-1α*, *TNF*, and *NF-κB*), lipid metabolism (eg, *PPARα*), and hypoxic response (eg, *HIF-1α*). One of the most important genes predicted by GSEA leading edge analysis, *STAT1*, also appeared high on the list of upstream activators. One of the candidate genes, *FAS*, involved in control of apoptosis, appeared high on the list but narrowly missed significance *Z*-score threshold (*Z* score, 1.981; see Table III.II in the online-only Data Supplement). IPA-predicted downstream cellular functions were also in broad agreement with GSEA results. The predicted increased downstream cellular functions can be categorized as (1) cellular movement; (2) immune cell trafficking and inflammatory response; (3) cell cycle, proliferation, and mitogenesis; (4) cell death and survival; (5) cell-to-cell signaling and interaction; and (6) metal and mineral metabolism (calcium in particular; Table III.III in the online-only Data Supplement). In plaques with large lipid core (%lipid area on T2 map, >25%), IPA showed significant upstream activation of IFN pathways (Table III.V and III.VI in the online-only Data Supplement), again consistent with and support our GSEA results, providing cross-platform validation.

### Convergence Between Biologically Regulated (From mRNA-GSEA) and Genetically Implicated Pathways (From GWAS-MAGENTA)

Using the top 200 GSEA-enriched pathways in symptomatic plaque macrophages as a superset, we found 8 of these pathways significantly cross enriched in MAGENTA (GSEA vs MAGENTA; *P*<0.05; FDR, <0.25); conversely, using the top 30 MAGENTA-enriched pathways from human SNP variants associated with coronary artery disease and ischemic stroke, we found 5 of these pathways significantly cross enriched in GSEA (MAGENTA vs GSEA; *P*<0.05; FDR, <0.25; Figure 6). Patterns of convergence emerged as 3 of 8 pathways in the GvsM analysis, and 3 of 5 pathways in MvsG analysis are directly concerned with lipid metabolism. By cross-referencing the leading edge subset of genes in both GvsM and MvsG analyses, an overlap of 10 genes was found. These included *ABCA1*, *ABCG1*, *APOC1*, *APOE*, *APOL2*, *BMP1*, *CETP*, *PLTP*, *PRKAA1*, and *SREBF1*. In other words, these overlapping pathways and their component genes are both differentially regulated in macrophages from symptomatic plaques and have been implicated in pathways associated with complications of atherosclerosis in GWAS. *ABCA1* and *APOE*, identified through this unbiased screen, were most significantly cross-enriched, being present, respectively, in 5 and 6 overlapping pathways, whilst themselves genes associated with known GWAS risk variants (Figure 6; also see Table IV in the online-only Data Supplement for the 314 SNPs used for MAGENTA).

## Discussion

Transcriptomic analyses of macrophages obtained from symptomatic vs asymptomatic human carotid plaques showed differential regulation of gene sets contributing to multiple functional pathways that clustered in cellular processes related to inflammation, lipid metabolism, response to hypoxia, cell proliferation, apoptosis, antigen presentation, and cellular energetics. Rather than focusing on differential single-gene expression, we employed GSEA. This approach conveys a number of advantages when compared with single-gene methods.^27,28^ First, it provides a structure for interpretation by identifying pathways and processes. Rather than focusing on highly regulated single genes (which can be difficult to interpret mechanistically), GSEA focuses on gene sets, which tend to be more reproducible and more interpretable. Second, when the members of a gene set exhibit strong cross-correlation, GSEA boosts signal-to-noise ratio making it possible to appreciate the contributions of even modest changes in individual genes. Third, leading edge analysis can help define gene subsets to elucidate key players and identify critical biological processes. Fourth, by also employing MAGENTA, it allows ready comparison of pathways and processes that might be implicated by SNPs occurring in both introns and exons, which can also be grouped in functional pathways.^30^

Previous studies of gene expression in human carotid plaques focused on differences in gene expression between stable and unstable segments of plaques. They have not analyzed any particular cell type,^17^ and gene expression has not been related to other important plaque morphological features, for example, size of the LRNC.^34^ Others have examined exclusively symptomatic plaques.^35^ In unsupervised analysis, we found that the symptomatic status of the plaque was associated with differential gene expression in macrophages but that the site of origin within the plaque was not. We, therefore, focused subsequent analyses on the former comparison. However, the presence of cholesterol/LRNC in plaque is an important determinant of both macrophage function and propensity to cause acute clinical events. To take this relationship into account, we also undertook a systematic quantification of plaque lipid using in vivo T2 mapping with MRI.^25^

The 7 categories of cellular functions identified by GSEA included both expected and novel biological insights. Plaque inflammation has long been shown to be a potent driver for atherosclerosis progression and thrombotic complications.^36^ In the current study, both inflammation and hypoxic responses were upregulated in macrophages derived from symptomatic plaques. In addition, processes related to cellular energetics, such as the Krebs (tricarboxylic acid) cycle and mitochondrial electron transport chain, were enhanced in macrophages from symptomatic plaques. This not only implies the increased metabolic demand of active inflammatory cells but highlights the potential intersection or cross talk between metabolic and inflammatory pathways that might be amenable to therapeutics that simultaneously target each of these processes.^37,38^ Importantly, genes associated with apoptosis were upregulated in symptomatic plaque-derived macrophages, along with a concomitant enhancement of pathways associated with cell proliferation. This is a novel finding in humans and is in agreement with a recent report in mice suggesting that although monocyte recruitment is the main mechanism of macrophage accumulation in early atherosclerosis, local proliferation of lesional macrophages dominates advanced atherosclerotic lesions.^39^ In contrast, our GSEA result showed a significant upregulation of the pathway involved in the termination of O-glycan biosynthesis in asymptomatic plaque-derived macrophages (*P*<0.001; FDR, 0.014). Adhesion molecules, such as the selectins, are examples of glycoproteins essential in mediating initial leukocyte-endothelial-cell adhesion events in atherosclerosis. O-glycosylated proteins, such as PSGL-1 (P-selectin glycoprotein ligand-1), are expressed by differentiated macrophages.^40^ Alteration or termination of the glycosylation process or glycan biosynthesis is, therefore, highly plausible, and likely to be relevant, in the activation or differentiation state of macrophages, which might suppress leukocyte recruitment in asymptomatic plaques.

Macrophages from plaques with high lipid content, quantified by T2 mapping with magnetic resonance, differentially activated inflammatory pathways and, in particular, *IFN*/*STAT1* pathways. Both type I and type II IFN pathways were activated with increasing plaque lipid content. *IFN-α* pathways were most significantly enriched in macrophages with the highest lipid content. While type I IFNs (IFN-α/β) are better known in response to viral infection,^41^ and type II IFN (IFN-γ) being more well known to be proatherogenic,^42^ a recent study has shown that *IFN-α* priming of THP1 cells induced increased oxLDL uptake and promoted macrophage foam cell formation.^43^ A growing body of evidence supports a role for the type I IFN (IFN-α/β) in promoting foam cell formation and atherogenesis,^44^ and novel biologic therapy is currently in development targeting *IFN-α/β* receptor.^45^

Macrophages produce IL-1β, which is promoted by the activation of NLRP3 inflammasomes by the formation of crystals as cellular free cholesterol accumulates.^46^ IL-1β and IL-1α exert proinflammatory effects that are inhibited by the endogenous antagonist IL-1RA (IL-1 receptor antagonist). Atherosclerosis-prone mice that are deficient for IL-1β develop smaller lesions,^47^ and administration of IL-1RA reduces early atherogenesis in mice,^48^ whereas IL-1RA–deficient mice have shown increased atherosclerosis^49^ and vascular inflammation, associated with destruction of elastic tissues.^50^ In the current study, numerous downstream effectors of *IL-1β* (eg, *IRAK*, *NF-kB*, and *MyD88*) were highly enriched in symptomatic plaques. The demonstration of activation of *IL-1β*–associated pathways in humans reinforces the rationale of targeting *IL-1β* therapeutically and may help to explain the benefits of IL-1β inhibition in the CANTOS (Canakinumab Anti-Inflammatory Thrombosis Outcomes Study) of canakinumab in patients after acute coronary syndromes.^51^

GWAS have identified a rapidly growing set of (≈161 at the time of writing) loci associated with atherosclerotic vascular disease and its complications.^20,21,52^ To fully capitalize on the knowledge of these variants requires understanding of the biological effects, and cell type(s) of expression, of these causal variants.^22^ Although many known genetic variants affecting the risk of atherosclerotic vascular disease are associated with established classic risk factors, such as hypertension or hyperlipidemia, a large proportion of the uncovered genetic association may be implicated in biological processes of the vessel wall.^52^ Macrophages are highly represented in atherosclerotic plaque and are important determinants of plaque behavior in relation to complications of atherosclerosis. Cointerrogation of published GWAS resources and plaque macrophage transcriptomes identified *APOE* and *ABCA1* as genes that were both differentially upregulated in unstable lesions and implicated by GWAS. The ATP-binding cassette transporters *ABCA1* and *ABCG1* are major mediators of macrophage cholesterol efflux. Recent studies have shown that the efflux activities of *ABCA1* and *ABCG1* also modulate macrophage expression of inflammatory cytokines and chemokines.^53^ Macrophage- specific deletion of *ABCA/G1* has been associated with activation of inflammatory genes,^54^ failure of efferocytosis,^55^ increased apoptosis,^56^ and increased propensity to develop lipid-rich, potentially unstable lesions.^54^

Similarly, *ApoE*-deficient mice develop severe hypercholesterolemia and atherosclerosis. It had generally been assumed that systemic hypercholesterolemia is the major proatherogenic factor in *ApoE*-deficient mice. However, the observation that macrophage-specific reintroduction of *ApoE* into *ApoE*-deficient mice ameliorates lipid lesion formation independent of any effects on systemic lipoprotein levels suggests that *ApoE* also may be exerting local effects at the blood vessel wall.^57^ Therefore, in relation to the GWAS variants, it is highly plausible that altered/reduced function of *ApoE* or *ABCA/G1* may result in lesions that are at higher risk of being associated with an acute plaque event.

Given the large body of animal data on mechanisms of plaque biology, it is unsurprising that the current study did not identify previously unrecognized pathways. Rather, the value of the transcriptomic data reported here resides in the analysis of the relevant human cell type obtained from tissue sampling in the context of the disease of interest.^22^ It provides a convergence point for evidence from (1) unbiased transcriptome analysis, (2) human GWAS,^20,29^ and (3) previous targeted (including cell specific) knockout in atherosclerosis-prone mice.^57,58^

There are a number of limitations in the Immuno-LCM and gene expression analysis methods used in this study. First, because of the conflicting demand during Immuno-LCM between cell procurement specificity and RNA quality, the current protocol of Immuno-LCM utilized a guide slide approach; and as such, only larger clusters of plaque macrophages can be procured accurately. In plaques where macrophages were sparse or scattered, this method could not be used reliably. Furthermore, since decalcification readily destroys RNA, plaques that contain large calcium deposits were excluded from LCM experiments because cryosectioning was almost always impossible.

In addition, pathway analyses, such as GSEA, MAGENTA, and IPA, rely on databases of biological pathways curated from published literature. The greatest body of work performed in this area at present relates to the fields of cancer research, and as such, there is an overrepresentation of cancer-related pathways in the analysis. It is, therefore, possible that there could potentially be many other relevant biological processes as yet unidentified or underrepresented in existing databases. Finally, transcriptomic analysis only examined one facet of the functional genome—there exists a complex multilayer regulatory network involving miRNA, dynamics of transcript or protein degradation, as well as post-translation modifications that could alter the flux of metabolic pathways and hence final functional effects.^59^

In conclusion, Immuno-LCM gene expression profiling of plaque macrophages successfully identified important biological processes that are upregulated specifically in recently symptomatic plaques. Lipid metabolic pathways showed convergence between biologically regulated (from mRNA) and genetically implicated pathways (from GWAS), highlighting the crucial role of macrophages as one of the cell types that contribute to the vascular risks associated with GWAS variants. Combining noninvasive plaque imaging by MRI T2 mapping with Immuno-LCM gene expression profiling further identified unique activation of *IFN*/*STAT1* pathways in carotid atherosclerosis, which correlated with the volume of plaque lipid. This demonstrates potential therapeutic targets and provides a molecular handle to a noninvasive imaging biomarker, highlighting the complementary roles of imaging and omics in an integrated platform in stratified medicine and future drug discovery.

## Acknowledgements

We express our gratitude to Phil Townsend for his general laboratory support and to the support of the Oxford Acute Vascular Imaging Centre (AVIC).

## Sources of Funding

J.T. Chai was a clinical research training fellow supported by the Medical Research Council and the Stroke Association UK (grant MR/K00266X/1); N. Ruparelia was supported by a British Heart Foundation (BHF) Clinical Research Fellowship; L. Biasiolli was supported by BHF (grant PG/15/74/31747); R.P. Choudhury was a Wellcome Trust senior clinical fellow. We also acknowledge the support of the National Institute for Health Research Oxford Biomedical Research Centre, the BHF Centre of Research Excellence (RE/13/1/30181), Oxford, and the Wellcome Trust Core Award (090532/Z/09/Z). R.P. Choudhury, A. Goel, and H. Watkins acknowledge the support of the Tripartite Immunometabolism Consortium, Novo Nordisk Foundation (grant NNF15CC0018486). A. Goel and T. Kyriakou acknowledge support from CVGenes@Target (grant HEALTH-F2-2013–601456) and Wellcome Trust institutional strategic support fund.

## Disclosures

None.

## Supplementary Material

## Nonstandard Abbreviations and Acronyms

CANTOS: Canakinumab Anti-Inflammatory Thrombosis Outcomes Study
CD: cluster of differentiation
FDR: false discovery rate
GSEA: gene set enrichment analysis
GWAS: genome-wide association study
IFN: interferon
IL: interleukin
IL-1RA: IL-1 receptor antagonist
Immuno-LCM: immunohistochemistry-guided laser capture microdissection
IPA: ingenuity pathway analysis
LCM: laser capture microdissection
LRNC: lipid-rich necrotic core
MAGENTA: meta-analysis gene set enrichment of variant associations
MRI: magnetic resonance imaging
PSGL-1: P-selectin glycoprotein ligand-1
RIN: RNA integrity number
TNF-α: tumor necrosis factor-α
