# Supplementary material for: Differential Gene Expression in Macrophages From Human Atherosclerotic Plaques Shows Convergence on Pathways Implicated by Genome-Wide Association Study Risk Variants
Source: Arterioscler Thromb Vasc Biol. 2018 Sep 6;38(11):2718–30. doi: 10.1161/ATVBAHA.118.311209 (PMC6217969; doi:10.1161/ATVBAHA.118.311209)
Supplement: Supplementary file 1 [file atv-38-2718-s001.pdf]

Major Resources Tables

Antibodies

Target antigen	Vendor or Source	Catalog #	Working concentration	Lot # (preferred but not required)
Mouse anti-human CD68 (clone KP-1)	Dako, Cambridge UK	M0814	1:150 (200 µg/ml)	
Mouse anti-human alpha-actin (clone 1A4)	Dako, Cambridge UK	M0851	1:100 (50 µg/ml)	
Rabbit anti-human Ki-67 (polyclonal)	Abcam, Cambridge, UK	Ab15580	1:100 (100 µg/ml)	
AF 488 Donkey anti-rabbit secondary antibody	Invitrogen	A-21206	1:1000 (2 µg/ml)	
AF 594 Goat anti-mouse secondary antibody	Invitrogen	A-21125	1:1000 (2 µg/ml)	

Quantitative real-time PCR primer

Gene symbol	Vendor	Assay ID
LEP	Life Technologies / Invitrogen	Taqman Assay ID: Hs00174877_m1
FABP4	Life Technologies / Invitrogen	Taqman Assay ID: Hs01086177_m1
CD163	Life Technologies / Invitrogen	Taqman Assay ID: Hs00174705_m1
HMOX1	Life Technologies / Invitrogen	Taqman Assay ID: Hs01110250_m1
TIMP3	Life Technologies / Invitrogen	Taqman Assay ID: Hs00165949_m1
MMP1	Life Technologies / Invitrogen	Taqman Assay ID: Hs00899658_m1
ADAMTS8	Life Technologies / Invitrogen	Taqman Assay ID: Hs00199836_m1
RGS5	Life Technologies / Invitrogen	Taqman Assay ID: Hs01555176_m1
GAPDH	Life Technologies / Invitrogen	Taqman Assay ID: Hs03929097_g1
