# Supplementary material for: Differential Gene Expression in Macrophages From Human Atherosclerotic Plaques Shows Convergence on Pathways Implicated by Genome-Wide Association Study Risk Variants
Source: Arterioscler Thromb Vasc Biol. 2018 Sep 6;38(11):2718–30. doi: 10.1161/ATVBAHA.118.311209 (PMC6217969; doi:10.1161/ATVBAHA.118.311209)
Supplement: Supplementary file 2 [file atv-38-2718-s002.pdf]

SUPPLEMENTAL MATERIAL

Supplemental methods

(i) Human carotid samples and quality control
Plaque was briefly inspected and divided at the level of maximal stenosis, with the proximal half of the plaque (including the bifurcation as anatomical landmark) processed for paraffin embedding for standard H&E and Masson trichrome histology; the distal halves were immediately snap frozen in Optimal Cutting Temperature (OCT) medium (VWR, Lutterworth, Leicestershire, UK) using crushed dry-ice ethanol slurry and stored in -80 ^{O}C for batch immunohistochemistry-guided LCM (immuno-LCM). Time for inspection and specimen preparation was kept to a minimum to avoid RNA degradation. To minimize technique variation and maintain quality control, 2 early plaque samples were used as internal reference to check RNA quality using Agilent Bioanalyzer at 6 monthly intervals to ensure minimal RNA degradation. RNA Integrity Number (RIN) variations on these quality checks were ≤ 0.5 RIN value throughout the duration of the study.

Immuno-LCM was attempted in 32 plaques (16 symptomatic and 16 asymptomatic), of which 4 plaques were excluded at the guide-slide preparation stage due to excessive calcification and /or lack of macrophage clusters. Immuno-LCM was completed for 28 plaques (15 symptomatic and 13 asymptomatic). Four (14%) plaques had RNA quality below the minimal threshold of RIN > 5.0 and were excluded from downstream analysis. The remaining 12 symptomatic and 12 asymptomatic plaques successfully progressed through amplification and microarray experiments. Not all plaques contain both core and cap macrophages. Only two plaques in the symptomatic group (2 out of 12), and two plaques in the asymptomatic group (2 out of 12), successfully underwent LCM procurement of both core-associated and cap-associated macrophages of sufficient RNA quality. The remaining plaques only had one (either core-associated, or cap-associated) type of macrophages harvested for downstream microarray. The 2x2 sample grid was shown below.

	Core-associated macrophages	Cap-associated macrophages	**Total**
Symptomatic	7	5	12
Asymptomatic	5	7	12
**Total**	12	12	24

The RINs were similar between macrophages procured from symptomatic and asymptomatic plaques (6.26 $\pm$ 1.07 vs. 6.49 $\pm$ 0.93, not significant). The average pre-LCM sample quality control RIN was 7.27 $\pm$ 0.96. The average RIN drop attributable to immuno-LCM protocol was 1.05 ± 1.03 (mean ± SD).

(ii) Optimisation of immunohistochemistry-guided LCM
In order to procure plaque macrophages and smooth muscle cells (as internal control) for downstream analysis, an immuno-LCM protocol for capturing CD68-positive and alpha-actin-positive cells was developed. Preliminary experiments demonstrated that direct immunostaining on LCM sections, despite adhering to meticulous decontamination precaution and using RNase inhibitors in all aqueous solutions, severely degraded RNA quality (average RIN < 3.0). This was thought to be caused by re-activation of intrinsic RNases within macrophages in any aqueous environment during staining. A “guideslide” approach was therefore adapted and modified from Feig, *et al.*[4] In order to discriminate cells based on immuno-phenotype

and micro-anatomical location, one frozen section (15 μm) was stained for Masson trichrome for plaque morphology, and 2 immediately adjacent frozen sections (15 μm) were stained with primary antibodies against CD68 and smooth muscle alpha actin in a rapid immuno-staining protocol.

(iii) Rapid immunohistochemistry for LCM "guideslide"
Cryosections were air-dried in room temperature for 5 minutes. They are then fixed in ice-cold acetone for 5 minutes. Slides were re-hydrated in PBS-T (PBS-Tween 20, 0.5%), and blocked with 0.3% hydrogen peroxide (Sigma Aldrich, Poole, UK) for 10 minutes. After further washing in PBS-T, slides were blocked with serum-free blocking solution (Dako, Cambridge, UK) for 10 minutes. Excess blocking solution was drained and slides immediately incubated with primary antibodies diluted with 2.5% normal horse serum for 10 minutes (Vectorstain ABC Universal Quick Kit – RTU (PK-7800), Vectorlab, Peterborough, UK). Slides were then washed with PBS-T and incubated with pre-diluted secondary antibody and pre-diluted ABC reagent, respectively, for 5 minutes each as per manufacturer's instructions. Peroxidase substrate visualization was performed using ImmPACT DAB (SK-4105, Vectorlab, Peterborough, UK) for up to 2 minutes. Slides were then counter-stained with haematoxylin (Sigma Aldrich, Poole, UK), washed, dehydrated in graded alcohol, and mounted. Primary antibodies used include mouse anti-human CD68 in 1:150 dilution and mouse anti-human alpha-actin in 1:100 dilution, both at room temperature (See Major Resources Table). Total preparation time for the guideslides using the above protocol is 60 minutes from cryosection and mounting.

The trichrome stained and immuno-stained sections were then used as templates, or "guideslides", to navigate and perform LCM on the next (up to) 15 serial LCM sections (15 μm each). This rapid IHC protocol is essential for the successful application of the "guideslide" LCM approach. This is because the specimen has to remain mounted to the specimen head within the cryostat during the entire duration of staining and subsequent LCM in order to maintain absolute precision in position and orientation of each of the subsequent serial sections. It would otherwise be impossible with conventional overnight immunohistochemistry protocol as the specimen orientation would have changed during specimen mounting / dismounting from cryostat.

(iv) Laser capture microdissection using Zeiss PALM Microbeam system
Nuclease-free PEN MembraneSlide™ (Carl Zeiss GmbH, Germany) were pre-cooled inside the cryostat chamber down to -20°C. Up to three 15 μm cryosections were thaw-mounted on each MembraneSlide™ by warming up small areas on the pre-chilled slide on the opposite side of the slide using a gloved finger. Each thaw-mounted section would spontaneously re-freeze within 30 seconds as the slide air-dried within the cryostat chamber. This is to ensure the minimum amount of thaw-time (and minimal degree of RNase reactivation and RNA degradation) before the sections were fixed and dehydrated. LCM slides were kept inside the dry air in the cryostat chamber at -20°C until ready and each slide was *individually* processed following the modified Zeiss LCM protocol with cresyl violet nuclear staining.

(v) Cresyl violet stain for individual LCM PEN MembraneSlide™
Cresyl violet staining solution was prepared fresh by dissolving 0.5g of cresyl violet (Sigma Aldrich, Poole, UK) in 50% ethanol and filtered before use. All solutions used were made with DEPC-treated Milli-Q water. Each LCM PEN MembraneSlide™ was *individually* fixed in ice-cold 70% ethanol for 2 minutes; followed by staining with ice-cold cresyl violet staining solution (prepared above) for 2 minutes. Each slide was then briefly rinsed in ice-cold 70% ethanol for a few dips, and quickly washed and dehydrated in ice-cold 100% ethanol with a few more dips. Slides were air-dried in a

cell culture grade hood until completely dry. Once completely dehydrated, RNA is stable in room temperature for at least a few hours (validation data available from Carl Zeiss Microscopy, Germany). Cresyl violet was chosen as the nuclear stain of choice because it is entirely alcohol-based; so staining with cresyl violet allows minimal exposure to an aqueous environment, which would otherwise reactivate intrinsic RNases within macrophages. In theory, RNA degradation starts the moment an LCM slide is removed from the cryostat chamber and goes through the staining process; therefore, as a quality control measurement the time taken from the beginning of cresyl violet stain to post-LCM cell lysis (stain-to-lysis time) was recorded. The median stain-to-lysis time for each LCM sample was 1 hour (range 30 minutes to 2 hours). Using this protocol there was minimal RNA degradation with an average RIN drop attributable to immune-LCM protocol of 1.05 ± 1.03.

(vi) Principal component analysis

Unsupervised principal component analysis (PCA) was performed, which showed a small group effect when comparing 'symptomatic' and 'asymptomatic' plaques [shown in main Figure 2A]; however, there was no separation when comparing 'core' and 'cap' macrophages ("Core" vs "Cap" PCA, shown below – yellow represents core and green represents cap).

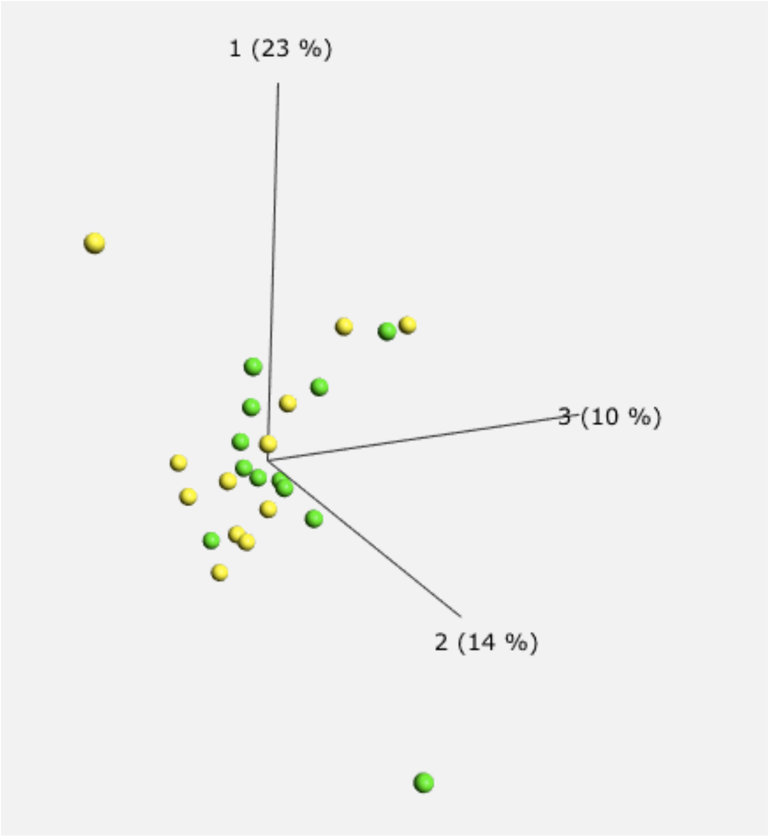

(vii) Ingenuity® Pathway Analysis

To further explore the upstream effectors and downstream functional consequence of the LCM-transcriptomic microarray findings, and to assess cross-platform agreement and consistency of the bioinformatic analysis, differentially expressed gene lists were uploaded to Ingenuity Pathway Analysis (IPA) software (Qiagen, Silicon Valley, Redwood City, CA, USA).

The upstream regulator analysis is based on prior knowledge of expected effects between transcriptional regulators and their target genes stored in the Ingenuity® Knowledge Base, which takes into account the potential transcription factor regulatory sites amongst the up/down regulated genes in the candidate differential expression gene list, and their direction of change, and predict the cascade of upstream transcriptional regulators that can explain the observed changes in gene expression in a given biological state.

The analysis generates two measurements, an "overlap P-value", which is calculated using Fisher's Exact Test to measure statistically significant overlap between the dataset genes and the genes regulated by a transcriptional regulator; and an "activation z-score", which infer the activation states of the predicted transcriptional regulators. A z-score of >2 or <2 is considered significant.

IPA downstream effects analysis, on the other hand, identifies cellular functions that are anticipated to increase or decrease given the observed gene expression changes. It is based on expected causal effects between genes and functions and are derived from the literature in the Ingenuity® Knowledge Base, which take into consideration the directionality of the observed gene expression changes.

IPA analysis was performed using differentially expressed genes with higher expression in macrophages from symptomatic plaques *vs.* asymptomatic plaques ($P < 0.05$ and fold-change >1.4); as well as from plaques with large lipid core (≥ 25% *vs.* < 25% Lipid Area on MRI T2 map; $P < 0.05$ and fold-change > 1.5).

(viii) Comparison between GSEA (mRNA-level) and MAGENTA (GWAS-level) data
The basis of evaluation in cross-enrichment is that both GSEA and MAGENTA work from the same gene set collections from the molecular signature database (http://www.broadinstitute.org /gsea/msigdb). At a conventional threshold of $P < 0.05$ and FDR < 0.25,[28] 379 pathways (from C2 and C5 collections) were significantly enriched in symptomatic plaque macrophages on GSEA based upon transcriptome (mRNA) level information. We selected 143 pathways that were also curated under Biocarta, Kegg, PID, and Reactome databases due to their wider application outside of GSEA / MSigDB. These combined with the 93 significantly enriched ($P < 0.05$, FDR < 0.25) C5 pathways gave a total of 236 pathways. The top 200 of these, ranked by P-values, were then selected. These top 200 enriched pathways were selected in order to construct a "superset" of a manageable size containing 10,723 individual genes. This "superset" was then used to run MAGENTA (GSEA results *versus* MAGENTA, "GvsM" comparison) against the 314 human SNPs with susceptibility association to coronary artery disease and ischaemic stroke.[19, 27]

Likewise, 53 pathways (from C2 and C5 collections) were significantly enriched from the 314 SNPs on MAGENTA based upon GWAS-level information. We then excluded 7 Ingenuity pathways (not available for GSEA), and 9 Panther pathways to create a similar set of pathways mirroring GSEA above (containing Biocarta, Kegg, PID, and Reactome pathways) to give a total of 37 pathways. The top 30 of these, ranked by P-values, were selected. These top 30 enriched pathways were selected in order to construct a similar "superset" of a manageable size containing 4,854 individual genes. This second "superset" was used to run GESA (MAGENTA results *versus* GESA, "MvsG" comparison) against the transcriptomic information from the symptomatic plaque macrophages. The overlap, or cross-enrichment, between "GvsM" and "MvsG" reflects the core set of pathways or biological processes that are both differentially regulated in macrophages from symptomatic plaques and have been implicated in pathways thought to mediate risk associations with complications of atherosclerosis in GWAS.

(ix) Gene assignment for GWAS SNPs in MAGENTA

The gene assignment is done by MAGENTA in a systematic non-biased manner [Segre AV, PLoS Genet 6(8): e1001058 (2010)]. Each gene is assigned a set of SNPs based on physical location (110kb upstream & 40kb downstream of transcript). The gene is then assigned a score ($P_g^{BestSNP}$) based on association p-values. Confounders like gene size, SNPs per kb, recombination hotspots, linkage disequilibrium, genetic distance is taken into account to optimize the gene score p-value. There are limitations as the SNPs which are beyond the 110kb upstream and 40kb downstream boundary are not considered which may have a distal transcriptional regulatory effect. MAGENTA assigned the 314 SNPs to a total of 642 genes. The distribution of the p-values of the gene score assignment is provided below.

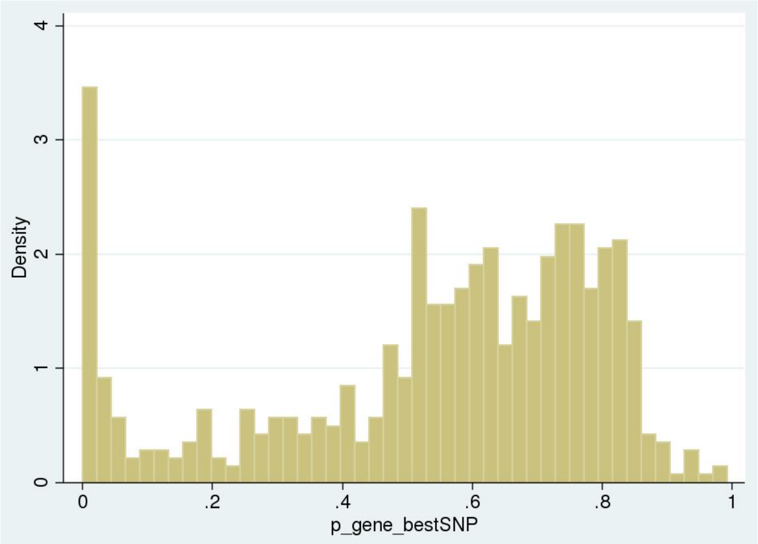

The eQTLs/functional lookup for GWAS SNPs corresponding to the cross-enriched genes (rs7412 is assigned to 2 genes hence only 3 rows) are provided by the table below (along with the distribution of the p-values of the gene score assignment above). For CETP gene, the proxy SNP's effect allele (T) is deleterious in CAD (OR=1.03) and shows increased expression (positive beta) of CEPT gene in Liver. The other 2 SNPs are non-synonymous (rs7412) or its good proxy (rs2066714 for ABCA1).

GWAS SNP	MAGENTA GENE ASSIGNMENT	MAGENTA GENE_Pvalue	CAD Effect Allele	CAD p-value	SNP	SNP type	r2	Effect Allele	CAD OR	eQTL Beta	eQTL p	Tissue	eGene
rs7412	APOE/APOC1	0.046/0.038	C	2.17E-19	rs7412	non-synonymous	1	Arg176Cys	1.154				APOE
rs247616	CETP	0.395	C	1.01E-06	rs36229491	eSNP	0.97	T	1.03	0.423	1.43E-11	Liver	CETP
rs4149311	ABCA1	0.377	T	9.06E-06	rs2066714	non-synonymous	0.88	Ile883Met	1.029				ABCA1

(x) Immunohistochemistry for Ki67 and confocal microscopy

Freshly cut, air-dried, cryosections were fixed in ice-cold acetone for 10 minutes, washed with PBS-Tween, and blocked with serum-free protein blocking solution (Dako, Cambridge, UK) for 2 hours in room temperature. They were then incubated with a primary antibody at 4°C overnight. After washing, cryosections were incubated with a secondary antibody at room temperature for 1 hour (see Major Resources

Table for primary and secondary antibodies used). After washing, cell nuclei were counterstained with Slowfade anti-fade reagent with DAPI (Invitrogen, Paisley, UK). Images were then taken using a Leica DM2500 microscope with QImaging MicroPublisher 5.0 RTV image sensor. Images were analysed using ImagePro Plus version 6.1 (MediaCybernetics, Silver Spring, MD, USA). Confocal fluorescence microscopy was performed using a Leica SP-5 laser-scanning confocal microscope fitted with a 63x 1.3NA oil-immersion objective (Zeiss, Cambridge, UK). Images were processed using Leica LAS AF Lite software and analysed using ImagePro Plus version 6.1 (MediaCybernetics, Silver Spring, MD, USA).

To ensure antibody binding specificity, appropriate IgG isotype control stainings were performed for monoclonal primary antibodies. For polyclonal primary antibodies, the peptides used to raise the antibodies were purchased from the antibody manufacturer for competitive peptide blocking experiment. This was performed by pre-incubating the polyclonal antibody with its raising peptide in a 1:20 ratio for 1 hour at room temperature before application of this mixture in the primary antibody incubation step described above.

Supplemental data

(i) Cell procurement is highly specific with immuno-LCM

To ascertain cell procurement specificity, macrophages and smooth muscle cells identified using the immuno-LCM protocol for this study were analysed using quantitative RT-PCR to check for specificity. Differential expression of CD68 and α-actin genes was used to assess cell procurement specificity. First, it was shown that CD68 transcript was selectively enriched in laser-captured cells from plaque macrophage (CD68 immuno-positive) area compared to whole tissue sections [Figure I-A]. Subsequently, LCM was used to separately procure cells from plaque macrophage (CD68 immuno-positive) area and smooth muscle cell (smooth muscle α-actin immuno-positive) area from the same plaque samples. Figure I-B shows the differential enrichment of CD68 and α-actin transcripts in cells from their respective LCM procurement area. Cells procured from plaque core macrophage regions showed a 3-fold higher expression of CD68 gene; whereas cells procured from plaque cap smooth muscle cells region showed a 5-fold higher expression of α-actin genes. This confirmed that the immuno-LCM protocol used in this study was highly capable of procuring different cell types with high degree of specificities.

(ii) Microarray validation using quantitative RT-PCR

Since the quantity of RNA extracted from immuno-LCM was very small, RNA was subjected to amplification prior to microarray experiment. To ensure RNA amplification maintained high fidelity and linearity, single gene quantitative RT-PCR was carried out on the original, *unamplified*, RNA for 8 genes (N = 6 per group) for both symptomatic *vs.* asymptomatic, and for cap *vs.* core comparisons [Figure II-A and II-B]. Genes were chosen based on the microarray data with those representing a significant fold-change (>1.5) between groups. Validation PCR confirmed good correlation between microarray results and single gene quantitative RT-PCR.

(iii) Additional MAGENTA analysis

The original MAGENTA analysis in the manuscript used the SNPs list obtained from Suppl Table 7 in Nelson et al paper (Nat Genet. 2017;49:1385-1391) which were derived after performing conditional analysis on an extended panel of SNPs (FDR 5% list) that obtained a CAD association p-value < ~10^{-4}. This extended list gives us a better opportunity to interrogate the genome for pathways rather than looking at only genome-wide SNPs. We included 10 SNPs from METASTROKE paper to be inclusive of cerebrovascular events. This 314 SNP list is now included in Supplemental Table IV.

During the review process of the manuscript, van der Harst et al published further novel SNPs in Circ Res. 2018 Feb 2; 122(3): 433-443. We therefore performed an additional MAGENTA analysis using the 161 genome-wide significant loci reported in van der Harst paper (plus 10 METASTROKE loci) and the pathways that have a nominal P-val < 0.05 are listed in Supplemental Table V. The only overlapping pathway in the new MAGENTA analysis is the REACTOME LIPID DIGESTION MOBILIZATION AND TRANSPORT pathway, which we highlighted in yellow. We did not observe any novel overlap in the new GvsM analysis. Overall, the new additional MAGENTA analysis using SNPs from the van der Harst et al paper strongly supports and reinforces our original MAGENTA analysis that lipid metabolic pathways were significant cross-enriched.

Supplemental figures

Supplemental Figure I – Selective enrichment by LCM

Figure I-A shows selective enrichment of CD68 transcripts in plaque macrophage (CD68 immuno-positive) region compared to whole tissue section. Figure II-B further confirmed differential expression of CD68 and α-actin transcripts from cells procured from macrophage region *vs.* plaque cap SMC (smooth muscle cells; α-actin immuno-positive, not shown here) region.

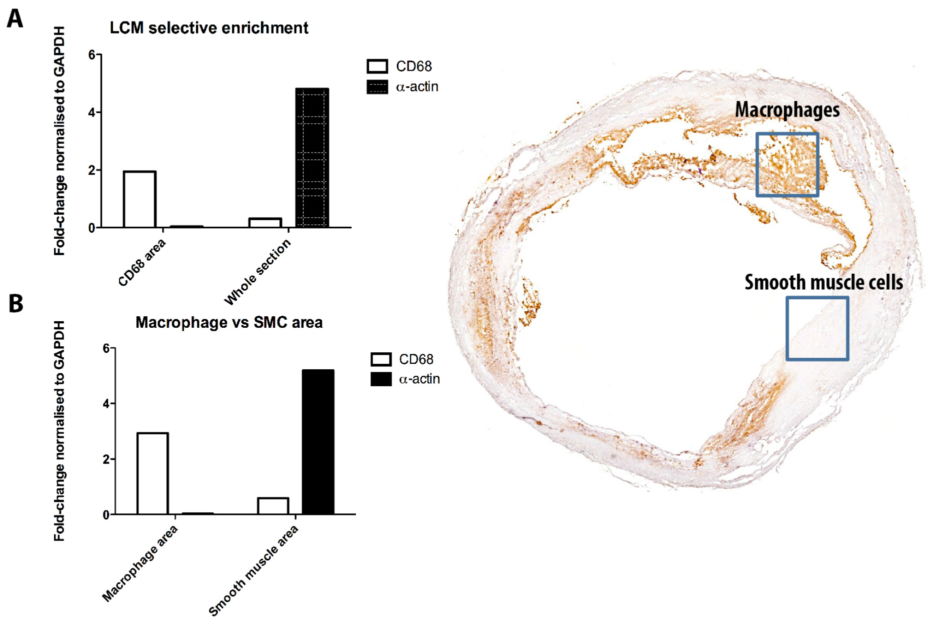

Supplemental Figure II - Single gene validation PCR of microarray experiment
Single gene PCR for 8 genes was performed on the unamplified RNA. The results showed good correlation with the microarray findings of the same biological samples. (N = 6 per group for qRT-PCR and 12 per group for microarray). Figure II-A shows comparison between symptomatic *vs.* asymptomatic samples; whereas Figure II-B shows comparisons between core *vs.* cap samples. HMOX-1: haemoxygenase-1. CD163: scavenger receptor cysteine-rich type 1 protein M130. RGS5: regulator of G-protein signaling 5. ADAMTS8: ADAM metallopeptidase with thrombospondin type 1 motif 8. FABP4: fatty acid binding protein 4. LEP: leptin. TIMP3: tissue inhibitor of metalloproteinases 3. MMP1: matrix metallopeptidase 1.

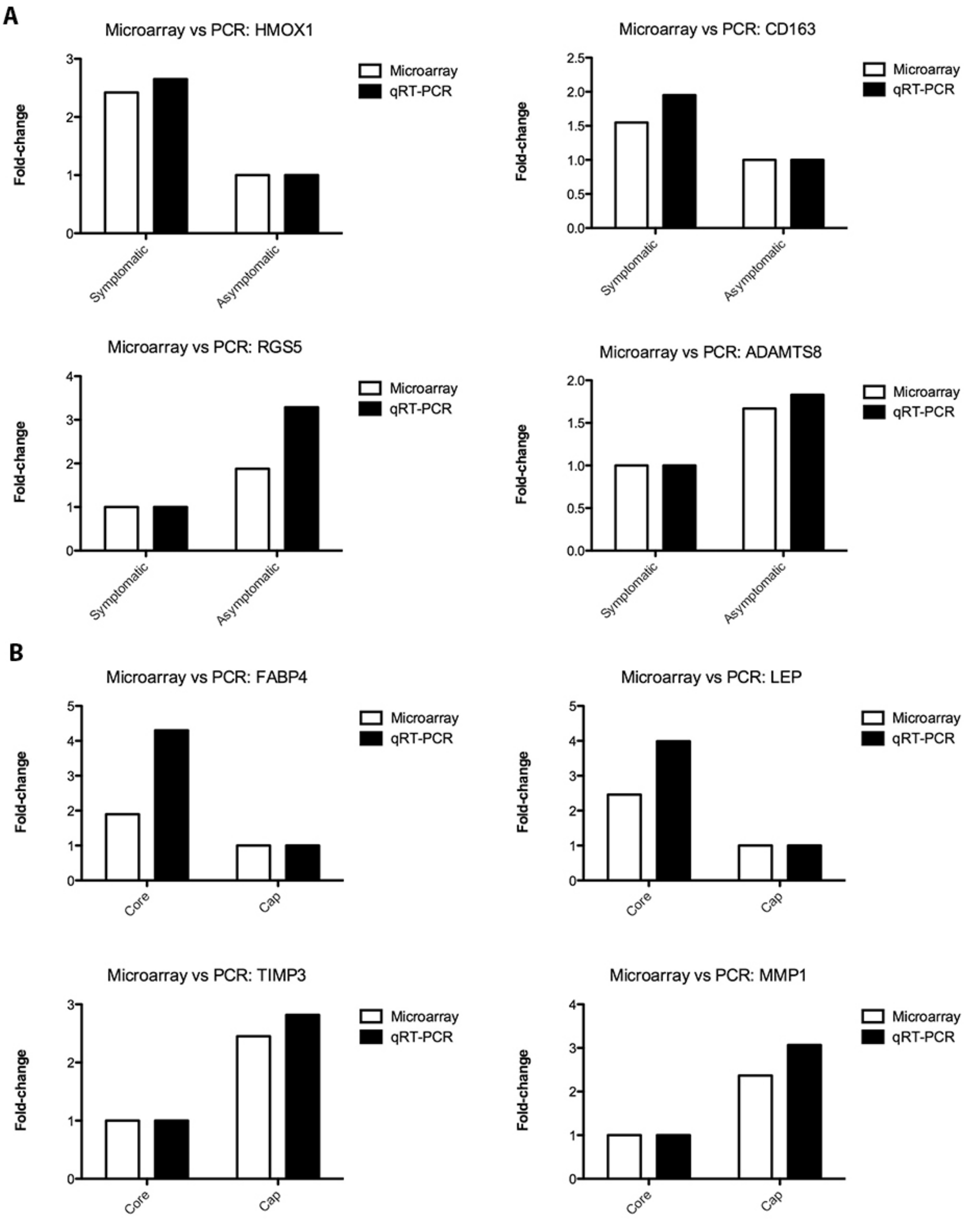

Supplemental tables

Table I.I GSEA analysis of enriched Hallmark collection pathways in symptomatic plaques. 14 Hallmark pathways significantly enriched in macrophages from symptomatic plaques. Statistical threshold set as P < 0.05, FDR Q < 0.25. NES = normalised enrichment score.

Pathway name	Size in gene set	NES	P-val	FDR Q-val
**HALLMARK_INTERFERON_GAMMA_RESPONSE**	199	1.656141	0.006012024	0.08919458
**HALLMARK_OXIDATIVE_PHOSPHORYLATION**	196	1.5656813	0.041353382	0.21178779
**HALLMARK_ADIPOGENESIS**	197	1.5446645	0.013833992	0.17851838
**HALLMARK_ALLOGRAFT_REJECTION**	200	1.5273249	0.009615385	0.12780593
**HALLMARK_FATTY_ACID_METABOLISM**	156	1.5214092	0.036679536	0.11646299
**HALLMARK_PEROXISOME**	102	1.516389	0.026871402	0.10459768
**HALLMARK_PROTEIN_SECRETION**	96	1.487225	0.039622642	0.12467822
**HALLMARK_GLYCOLYSIS**	198	1.4829834	0.038610037	0.11579916
**HALLMARK_COMPLEMENT**	199	1.4777089	0.015594542	0.11165891
**HALLMARK_IL2_STAT5_SIGNALING**	193	1.4598461	0.016032064	0.10866111
**HALLMARK_XENOBIOTIC_METABOLISM**	200	1.4596412	0.037924152	0.10038379
**HALLMARK_IL6_JAK_STAT3_SIGNALING**	87	1.4528576	0.033797216	0.10326559
**HALLMARK_HEME_METABOLISM**	197	1.4493978	0.02357564	0.09943583
**HALLMARK_APOPTOSIS**	161	1.3689339	0.042510122	0.13592671

Table I.II GSEA analysis of enriched pathways in symptomatic plaques.

379 C2 gene sets significantly enriched in macrophages from symptomatic plaques. Statistical threshold set as P < 0.05, FDR Q < 0.25. NES = normalized enrichment score.

Pathway name	Size in gene set	NES	P-val	FDR Q-val
**BANDRES_RESPONSE_TO_CARMUSTIN_MGMT_24HR_DN**	33	1.5144116	0.033797216	0.24937546
**REACTOME_ANTIGEN_PROCESSING_UBIQUITINATION_PROTEASOME_DEGRADATION**	206	1.5140097	0.026717557	0.24914505
**SARRIO_EPITHELIAL_MESENCHYMAL_TRANSITION_DN**	147	1.5139024	0.012	0.24810326
**PETROVA_PROX1_TARGETS_DN**	64	1.513802	0.016736401	0.24718794
**REACTOME_APC_C_CDC20_MEDIATED_DEGRADATION_OF_MITOTIC_PROTEINS**	69	1.5136893	0.03125	0.2463167
**REACTOME_S_PHASE**	101	1.5127031	0.035019454	0.24781145

DORN_ADENOVIRUS_INFECTION_12HR_UP	28	1.5124867	0.02008032	0.2471742
TSUNODA_CISPLATIN_RESISTANCE_DN	50	1.5124478	0.032128513	0.24602576
TAKAO_RESPONSE_TO_UVB_RADIATION_UP	85	1.5120571	0.011428571	0.2457558
SCHURINGA_STAT5A_TARGETS_UP	21	1.5118508	0.036217302	0.24498342
WENG_POR_TARGETS_LIVER_UP	38	1.5114162	0.003752345	0.24482584
TIEN_INTESTINE_PROBIOTICS_6HR_UP	54	1.5113629	0.014925373	0.24383962
POOLA_INVASIVE_BREAST_CANCER_UP	272	1.5105557	0.012024048	0.24515723
GENTILE_UV_HIGH_DOSE_UP	24	1.5104305	0.035714287	0.24435522
SESTO_RESPONSE_TO_UV_C1	69	1.5102129	0.03508772	0.2439206
DER_IFN_GAMMA_RESPONSE_UP	70	1.5097867	0.038610037	0.24402307
REACTOME_PROCESSING_OF_CAPPED_INTRONLESS_PRE_MRNA	22	1.5091698	0.017208412	0.24326351
REACTOME_TCA_CYCLE_AND_RESPIRATORY_ELECTRON_TRANSPORT	120	1.5088958	0.04085603	0.24288505
WAKABAYASHI_ADIPOGENESIS_PPARG_RXRA_BOUND_36HR	138	1.5084497	0.011605416	0.24298202
BYSTRYKH_HEMATOPOIESIS_STEM_CELL_QTL_CIS	120	1.508349	0.02851711	0.24210994
KAYO_AGING_MUSCLE_DN	121	1.5081569	0.030075189	0.24145012
SCHURINGA_STAT5A_TARGETS_DN	15	1.5078896	0.013133208	0.24111278
KYNG_RESPONSE_TO_H2O2_VIA_ERCC6_DN	46	1.5067153	0.01923077	0.24314699
GNATENKO_PLATELET_SIGNATURE	46	1.5066727	0.005758158	0.24098971
KEGG_SNARE_INTERACTIONS_IN_VESICULAR_TRANSPORT	38	1.5064203	0.015686275	0.24055682
REACTOME_INHIBITION_OF_THE_PROTEOLYTIC_ACTIVITY_OF_APC_C_REQUIRED_FOR_THE_ONSET_OF_ANAPHASE_BY_MITOTIC_SPINDLE_CHECKPOINT_COMPONENTS	22	1.5058587	0.034883723	0.24117362
MUNSHI_MULTIPLE_MYELOMA_UP	79	1.5053751	0.0227704	0.24156426
SESTO_RESPONSE_TO_UV_C0	106	1.5053335	0.022944551	0.24063353
MARKS_ACETYLATED_NON_HISTONE_PROTEINS	15	1.5049043	0.015009381	0.2407039
RASHI_RESPONSE_TO_IONIZING_RADIATION_6	79	1.5040202	0.007843138	0.24226007
REACTOME_RNA_POL_I_TRANSCRIPTION_INITIATION	25	1.5036091	0.036398467	0.24238
KYNG_ENVIRONMENTAL_STRESS_RESPONSE_UP	56	1.5027336	0.017612524	0.24168336
MELLMAN_TUT1_TARGETS_DN	47	1.5025938	0.007858546	0.24104062
LU_IL4_SIGNALING	90	1.5022625	0.013944224	0.2409626
YANG_BREAST_CANCER_ESR1_LASER_DN	48	1.5022347	0.017110266	0.23996416
AMUNDSON_GENOTOXIC_SIGNATURE	101	1.5013996	0.007692308	0.24125974
ALONSO_METASTASIS_UP	188	1.5013155	0.015503876	0.2403954
BYSTROEM_CORRELATED_WITH_IL5_DN	63	1.5001993	0.011811024	0.24148981
WINTER_HYPOXIA_METAGENE	232	1.4997333	0.015655577	0.24174967
BRUNO_HEMATOPOIESIS	64	1.4996777	0.003976143	0.24084347
LENAOUR_DENDRITIC_CELL_MATURATION_DN	127	1.4992911	0.01775148	0.24092096
BIOCARTA_DC_PATHWAY	22	1.4987441	0.049382716	0.241345
BURTON_ADIPOGENESIS_PEAK_AT_2HR	51	1.4985673	0.042424243	0.24090992
SENESE_HDAC2_TARGETS_UP	108	1.4985275	0.019193858	0.23996566
GAVIN_FOXP3_TARGETS_CLUSTER_T7	95	1.4968079	0.018656716	0.2427119
MOOTHA_TCA	16	1.4961965	0.007707129	0.24340804
REACTOME_TRAF6_MEDIATED_INDUCTION_OF_NFKB_AND_MAP_KINASES_UPON_TLR7_8_	72	1.4960093	0.007797271	0.24196623

OR_9_ACTIVATION				
VILIMAS_NOTCH1_TARGETS_DN	20	1.495908	0.030303031	0.24127449
FARMER_BREAST_CANCER_CLUSTER_7	19	1.4957123	0.021653544	0.24078323
KEGG_ALLOGRAFT_REJECTION	38	1.4955549	0.04989605	0.24020645
REACTOME_SYNTHESIS_OF_PIPS_AT_THE_GOLGI_MEMBRANE	17	1.4951563	0.022132797	0.24044634
ONDER_CDH1_TARGETS_1_DN	158	1.495079	0.015873017	0.23970631
CHEN_LVAD_SUPPORT_OF_FAILING_HEART_DN	42	1.4950229	0.018255578	0.23792985
RHODES_CANCER_META_SIGNATURE	62	1.4945414	0.015414258	0.2363265
REACTOME_E2F_MEDIATED_REGULATION_OF_DNA_REPLICATION	26	1.4943504	0.035433073	0.2358642
REACTOME_PHOSPHORYLATION_OF_THE_APC_C	20	1.4939685	0.046875	0.2358952
GESERICK_TERT_TARGETS_DN	20	1.4939443	0.036072146	0.23503676
DASU_IL6_SIGNALING_UP	58	1.4939307	0.011605416	0.23417188
GAZDA_DIAMOND_BLACKFAN_ANEMIA_PROGENITOR_UP	39	1.4937316	0.007751938	0.23292744
YIH_RESPONSE_TO_ARSENITE_C3	35	1.4936087	0.016096579	0.23245001
CHEN_LUNG_CANCER_SURVIVAL	27	1.4933848	0.022900764	0.23218825
REACTOME_DESTABILIZATION_OF_MRNA_BY_BRF1	17	1.4930067	0.033932135	0.23127273
TOMIDA_METASTASIS_UP	26	1.4929792	0.026768642	0.23047328
ZHONG_SECRETOME_OF_LUNG_CANCER_AND_FIBROBLAST	130	1.4929476	0.023076924	0.22966711
RUAN_RESPONSE_TO_TROGLITAZONE_DN	19	1.4921572	0.02584493	0.23068951
WENG_POR_TARGETS_GLOBAL_UP	19	1.4920156	0.02631579	0.23015365
FONTAINE_FOLLICULAR_THYROID_ADENOMA_DN	67	1.4919815	0.01980198	0.2294156
BIOCARTA_BCELLSURVIVAL_PATHWAY	15	1.4914824	0.021825397	0.22983897
CHANDRAN_METASTASIS_TOP50_UP	34	1.4913754	0.011560693	0.22933173
REACTOME_PREFOLDIN_MEDIATED_TRANSFER_OF_SUBSTRATE_TO_CCT_TRIC	26	1.491109	0.030131826	0.22747213
MA_MYELOID_DIFFERENTIATION_UP	39	1.4907746	0.025145067	0.22744882
NIKOLSKY_OVERCONNECTED_IN_BREAST_CANCER	22	1.49074	0.008	0.22668043
REACTOME_REGULATION_OF_HYPOXIA_INDUCIBLE_FACTOR_HIF_BY_OXYGEN	24	1.4905448	0.025242718	0.22641693
HILLION_HMGA1B_TARGETS	91	1.4899417	0.043643262	0.22712646
OKAMOTO_LIVER_CANCER_MULTICENTRIC_OCCURRENCE_UP	24	1.4896686	0.02259887	0.22696845
BIOCARTA_CHEMICAL_PATHWAY	22	1.489556	0.030425964	0.2263852
SHIN_B_CELL_LYMPHOMA_CLUSTER_5	17	1.489431	0.03629032	0.22589697
LI_AMPLIFIED_IN_LUNG_CANCER	177	1.4893279	0.03041825	0.22452997
TOOKER_GEMCITABINE_RESISTANCE_UP	76	1.4888519	0.025	0.22507975
LI_DCP2_BOUND_MRNA	86	1.4884146	0.037523452	0.22539687
BIOCARTA_STRESS_PATHWAY	25	1.4879861	0.01980198	0.22554268
HAHTOLA_MYCOSIS_FUNGOIDES_CD4_UP	64	1.4875959	0.03929273	0.22579724
JAIN_NFKB_SIGNALING	73	1.4875232	0.017110266	0.22513114
SPIRA_SMOKERS_LUNG_CANCER_UP	38	1.4872758	0.03646833	0.2250347
TAKAO_RESPONSE_TO_UVB_RADIATION_DN	97	1.4870822	0.02457467	0.22471853
LE_NEURONAL_DIFFERENTIATION_DN	19	1.4870613	0.043222003	0.22399001
KEGG_SYSTEMIC_LUPUS_ERYTHEMATOSUS	136	1.4870183	0.049115915	0.22334948
KAMIKUBO_MYELOID_CEBPA_NETWORK	28	1.4870132	0.048879836	0.22259767

CHIANG_LIVER_CANCER_SUBCLASS_UNANNOTATED_DN	182	1.4867957	0.04085603	0.22241662
YAO_TEMPORAL_RESPONSE_TO_PROGESTERONE_CLUSTER_9	73	1.4862001	0.011650485	0.22160402
DAIRKEE_TERT_TARGETS_UP	348	1.4861504	0.02457467	0.22094706
YAMAZAKI_TCEB3_TARGETS_DN	205	1.4861398	0.013513514	0.22023246
REACTOME_RNA_POL_II_TRANSCRIPTION	95	1.4860086	0.036190476	0.2198617
ZAMORA_NOS2_TARGETS_UP	68	1.485183	0.023483366	0.22046506
REACTOME_MRNA_PROCESSING	149	1.485126	0.030303031	0.21992205
POS_HISTAMINE_RESPONSE_NETWORK	32	1.4849325	0.023715414	0.21891688
HU_ANGIOGENESIS_DN	37	1.4845054	0.024667932	0.21925087
REACTOME_BIOSYNTHESIS_OF_THE_N_GLYCAN_PRECURSOR_DOLICHOL_LIPID_LINKED_OLIGOSACCHARIDE_LLO_AND_TRANSFER_TO_A_NASCENT_PROTEIN	29	1.4844391	0.014	0.21866849
PUIFFE_INVASION_INHIBITED_BY_ASCITES_DN	135	1.4841058	0.019011406	0.21795739
TAKEDA_TARGETS_OF_NUP98_HOXA9_FUSION_8D_DN	194	1.4839447	0.01178782	0.2176802
MORI_MATURE_B_LYMPHOCYTE_UP	87	1.4839203	0.015748031	0.2170306
BIOCARTA_MAPK_PATHWAY	86	1.4835997	0.03006012	0.21702048
REACTOME_LIPID_DIGESTION_MOBILIZATION_AND_TRANSPORT	44	1.4832237	0.013565891	0.21719252
RUTELLA_RESPONSE_TO_CSF2RB_AND_IL4_DN	300	1.4827158	0.019193858	0.2178182
XU_GH1_EXOGENOUS_TARGETS_DN	117	1.4825554	0.015873017	0.21753094
DAZARD_RESPONSE_TO_UV_NHEK_UP	235	1.4822882	0.020833334	0.21748675
LEI_MYB_TARGETS	312	1.4822828	0.011976048	0.21682101
IVANOVA_HEMATOPOIESIS_LATE_PROGENITOR	496	1.4820384	0.036398467	0.216793
SUNG_METASTASIS_STROMA_DN	51	1.4816551	0.031620555	0.21695335
WILLIAMS_ESR2_TARGETS_UP	27	1.481504	0.019723866	0.21663009
REACTOME_HDL_MEDIATED_LIPID_TRANSPORT	15	1.4815004	0.04780115	0.21596341
KEEN_RESPONSE_TO_ROSIGLITAZONE_UP	36	1.4814512	0.038387716	0.21540117
MONNIER_POSTRADIATION_TUMOR_ESCAPE_DN	334	1.4814088	0.015686275	0.2147971
REACTOME_CITRIC_ACID_CYCLE_TCA_CYCLE	23	1.4805017	0.019607844	0.21620697
SCHUHMACHER_MYC_TARGETS_UP	78	1.4802965	0.025096525	0.21608828
GERHOLD_ADIPOGENESIS_DN	64	1.4795368	0.019607844	0.21671641
OUELLET_OVARIAN_CANCER_INVASIVE_VS_LMP_UP	114	1.4793952	0.040229887	0.21638207
KIM_ALL_DISORDERS_DURATION_CORR_DN	140	1.4787568	0.029795159	0.2165559
BIOCARTA_CASPASE_PATHWAY	23	1.4784873	0.030181086	0.21659812
ROVERSI_GLIOMA_COPY_NUMBER_DN	53	1.4784226	0.035580523	0.21612366
ZHENG_RESPONSE_TO_ARSENITE_DN	18	1.4781826	0.0251938	0.21611792
BASSO_CD40_SIGNALING_UP	101	1.4780802	0.046875	0.2156953
DAZARD_UV_RESPONSE_CLUSTER_G1	65	1.4779023	0.04664179	0.21543191
REACTOME_PPARA_ACTIVATES_GENE_EXPRESSION	103	1.4775715	0.023076924	0.21573046
WANG_SMARCE1_TARGETS_DN	350	1.4775501	0.019193858	0.21512176
KEGG_HISTIDINE_METABOLISM	29	1.4775462	0.04	0.21449094
DAZARD_UV_RESPONSE_CLUSTER_G4	19	1.4772506	0.044921875	0.21404888
WILENSKY_RESPONSE_TO_DARAPLADIB	29	1.4768528	0.027504912	0.21359116

VIETOR_IFRD1_TARGETS	22	1.4765894	0.048262548	0.21354622
LUI_THYROID_CANCER_PAX8_PPARG_DN	43	1.4765024	0.042671613	0.21311542
MOREAUX_B_LYMPHOCYTE_MATURATION_BY_TACI_DN	66	1.4764687	0.038986355	0.21256477
QI_HYPOXIA	132	1.4762601	0.013833992	0.2125172
REACTOME_LYSOSOME_VESICLE_BIOGENESIS	23	1.4762306	0.025145067	0.21196736
REACTOME_PROTEIN_FOLDING	51	1.4760199	0.03409091	0.21183062
HUANG_GATA2_TARGETS_UP	140	1.4759978	0.026666667	0.21127923
REACTOME_INHIBITION_OF_VOLTAGE_GATED_CA2_CHANNELS_VIA_GBETA_GAMMA_SUBUNITS	25	1.4758431	0.02016129	0.21095425
REACTOME_MRNA_SPLICING_MINOR_PATHWAY	40	1.4757552	0.03088803	0.21054809
KEGG_BLADDER_CANCER	42	1.4755396	0.017612524	0.21040916
MOREAUX_MULTIPLE_MYELOMA_BY_TACI_DN	154	1.4754444	0.043643262	0.20945515
BIOCARTA_41BB_PATHWAY	17	1.4753077	0.047528517	0.20917158
DUTTA_APOPTOSIS_VIA_NFKB	32	1.475265	0.04085603	0.20868266
NEWMAN_ERCC6_TARGETS_UP	25	1.4751762	0.026052104	0.20824033
LEONARD_HYPOXIA	43	1.4751759	0.04183267	0.20766027
ZHAN_MULTIPLE_MYELOMA_SUBGROUPS	30	1.475007	0.023483366	0.20745635
CUI_TCF21_TARGETS_DN	29	1.4748987	0.03307393	0.20714368
LIU_VAV3_PROSTATE_CARCINOGENESIS_UP	87	1.4734627	0.02366864	0.20865281
BOGNI_TREATMENT_RELATED_MYELOID_LEUKEMIA_UP	29	1.4734343	0.023121387	0.20817469
REACTOME_TRIF_MEDIATED_TLR3_SIGNALING	69	1.4731424	0.01764706	0.20769478
VANTVEER_BREAST_CANCER_POOR_PROGNOSIS	49	1.4730688	0.012396694	0.20725554
WANG_CLIM2_TARGETS_DN	171	1.4727656	0.029296875	0.20730276
PARK_APL_PATHOGENESIS_DN	50	1.4726825	0.026465029	0.20690325
BIOCARTA_TNFR1_PATHWAY	29	1.4725682	0.03256705	0.20598906
MARTORIATI_MDM4_TARGETS_FETAL_LIVER_UP	206	1.4721305	0.011538462	0.2057992
LIM_MAMMARY_LUMINAL_PROGENITOR_UP	57	1.4719672	0.012096774	0.20557551
MOREIRA_RESPONSE_TO_TSA_DN	18	1.4712352	0.0331384	0.20616435
KAAB_FAILED_HEART_VENTRICLE_DN	41	1.4710413	0.042226486	0.20553333
BURTON_ADIPOGENESIS_9	88	1.4708221	0.023121387	0.20541517
SAKAI_CHRONIC_HEPATITIS_VS_LIVER_CANCER_UP	80	1.470796	0.04255319	0.20494291
PID_GMCSF_PATHWAY	37	1.4701467	0.021611001	0.20526472
CHEN_LIVER_METABOLISM_QTL_CIS	85	1.4695363	0.037181996	0.2054382
MEINHOLD_OVARIAN_CANCER_LOW_GRADE_DN	20	1.4690769	0.03875969	0.20599625
PODAR_RESPONSE_TO_ADAPHOSTIN_DN	16	1.4687729	0.02811245	0.20517322
FAELT_B_CLL_WITH_VH3_21_DN	48	1.4685446	0.030947777	0.2040882
MULLIGHAN_MLL_SIGNATURE_1_UP	359	1.4684162	0.027504912	0.20378599
HOLLEMAN_ASPARAGINASE_RESISTANCE_ALL_DN	23	1.4682978	0.035363458	0.20355381
REACTOME_ADP_SIGNALLING_THROUGH_P2RY1	25	1.4682935	0.031434186	0.2030452
GROSS_HYPOXIA_VIA_ELK3_AND_HIF1A_UP	136	1.4681828	0.007984032	0.20276564
BURTON_ADIPOGENESIS_1	33	1.467741	0.030947777	0.20314172
ACEVEDO_NORMAL_TISSUE_ADJACENT_TO_LIVER_TUMOR_DN	336	1.4676385	0.03809524	0.20285079

**WELCSH_BRCA1_TARGETS_DN**	135	1.4673189	0.039252337	0.20298167
**KYNG_RESPONSE_TO_H2O2**	71	1.4672376	0.007490637	0.20268361
**SEIDEN_MET_SIGNALING**	19	1.4671715	0.030947777	0.20233423
**HILLION_HMGA1_TARGETS**	89	1.467052	0.047348484	0.20213027
**GOLUB_ALL_VS_AML_UP**	24	1.4667897	0.01980198	0.20219159
**REACTOME_ASPARAGINE_N_LINKED_GLYCOSYLATION**	79	1.466692	0.037475344	0.20184667
**YAO_TEMPORAL_RESPONSE_TO_PROGESTERONE_CLUSTER_1**	67	1.4655514	0.02631579	0.20274721
**VALK_AML_CLUSTER_16**	23	1.4649193	0.028513238	0.20343277
**NADLER_OBESITY_UP**	61	1.4648751	0.011583012	0.20301926
**HOFFMANN_SMALL_PRE_BII_TO_IMMATURE_B_LYMPHOCYTE_UP**	69	1.4639871	0.034285713	0.2045973
**VANTVEER_BREAST_CANCER_ESR1_DN**	228	1.4637508	0.029013539	0.2040997
**GOLDRATH_HOMEOSTATIC_PROLIFERATION**	163	1.4634342	0.034548946	0.20387019
**WOOD_EBV_EBNA1_TARGETS_DN**	47	1.463171	0.033864543	0.20340283
**LANG_MYB_FAMILY_TARGETS**	29	1.4629971	0.02851711	0.2032227
**SYED_ESTRADIOL_RESPONSE**	19	1.4625384	0.020715632	0.20385297
**MARSON_FOXP3_TARGETS_UP**	63	1.4619178	0.011450382	0.20459151
**BROCKE_APOPTOSIS_REVERSED_BY_IL6**	139	1.4616885	0.017208412	0.2045624
**MCDOWELL_ACUTE_LUNG_INJURY_UP**	45	1.4614757	0.044573642	0.20455568
**GERY_CEBP_TARGETS**	125	1.4612154	0.013833992	0.2041349
**LIU_IL13_PRIMING_MODEL**	15	1.4611065	0.038986355	0.20388213
**RUAN_RESPONSE_TO_TNF_DN**	83	1.4609498	0.03256705	0.20374888
**DAIRKEE_CANCER_PRONE_RESPONSE_BPA_E2**	116	1.4608184	0.029850746	0.20214301
**KORKOLA_EMBRYONAL_CARCINOMA_UP**	37	1.460512	0.02952756	0.20231996
**KEGG_TOLL_LIKE_RECEPTOR_SIGNALING_PATHWAY**	100	1.4604472	0.039215688	0.20193464
**UEDA_CENTRAL_CLOCK**	85	1.4604089	0.013282732	0.20154794
**JIANG_VHL_TARGETS**	124	1.4602784	0.036679536	0.20086876
**JIANG_HYPOXIA_VIA_VHL**	31	1.4601492	0.039399624	0.20063289
**REACTOME_NUCLEOTIDE_LIKE_PURINERGIC_RECEPTORS**	15	1.4593568	0.027944112	0.20082903
**REACTOME_INSULIN_SYNTHESIS_AND_PROCESSING**	21	1.4593482	0.0234375	0.20037423
**TSAI_RESPONSE_TO_IONIZING_RADIATION**	148	1.4590073	0.01764706	0.20065059
**RASHI_RESPONSE_TO_IONIZING_RADIATION_1**	43	1.4583751	0.0415879	0.20050232
**BIOCARTA_ERK_PATHWAY**	28	1.4582645	0.03359684	0.20022093
**ZHAN_MULTIPLE_MYELOMA_DN**	41	1.4581132	0.030947777	0.20010114
**PID_CASPASE_PATHWAY**	51	1.4580185	0.01934236	0.19985874
**TARTE_PLASMA_CELL_VS_B_LYMPHOCYTE_DN**	37	1.4578419	0.02952756	0.19890516
**BIOCARTA_FAS_PATHWAY**	30	1.4577131	0.023391813	0.19872707
**BOYLAN_MULTIPLE_MYELOMA_PCA3_DN**	66	1.4575433	0.026717557	0.1986528
**LEE_METASTASIS_AND_RNA_PROCESSING_UP**	17	1.4571538	0.036750484	0.19903913
**HOLLEMAN_PREDNISOLONE_RESISTANCE_ALL_DN**	19	1.4569472	0.018903593	0.19894706
**ZHANG_RESPONSE_TO_CANTHARIDIN_UP**	18	1.4567795	0.034979425	0.19886063
**HINATA_NFKB_TARGETS_KERATINOCYTE_UP**	91	1.4564337	0.031558186	0.1986593
**SWEET_KRAS_ONCOGENIC_SIGNATURE**	88	1.4563377	0.023121387	0.19839925

IVANOVA_HEMATOPOIESIS_INTERMEDIATE_PROGENITOR	138	1.4563175	0.030245747	0.19801195
DUNNE_TARGETS_OF_AML1_MTG8_FUSION_UP	48	1.4562664	0.03742204	0.1976743
YAGI_AML_SURVIVAL	122	1.4558709	0.009652509	0.19807208
BIOCARTA_NKCELLS_PATHWAY	20	1.4556777	0.049115915	0.19768667
PID_BCR_5PATHWAY	65	1.455161	0.031496063	0.19785883
GERHOLD_ADIPOGENESIS_UP	49	1.4549737	0.04315197	0.19773614
SCHAEFFER_PROSTATE_DEVELOPMENT_6HR_UP	167	1.4549301	0.027237354	0.19740449
BIOCARTA_CD40_PATHWAY	15	1.4541649	0.028957529	0.19806807
BIOCARTA_RACCYCD_PATHWAY	26	1.4527202	0.023529412	0.20055285
MORI_LARGE_PRE_BII_LYMPHOCYTE_DN	57	1.4512357	0.02745098	0.2023926
IVANOVA_HEMATOPOIESIS_MATURE_CELL	268	1.4504292	0.03137255	0.20333493
CAFFAREL_RESPONSE_TO_THC_UP	31	1.4503958	0.033834588	0.20298463
CAFFAREL_RESPONSE_TO_THC_24HR_5_UP	31	1.4501041	0.033333335	0.2031656
SWEET_LUNG_CANCER_KRAS_UP	468	1.4491867	0.03088803	0.20433636
BIOCARTA_UCALPAIN_PATHWAY	18	1.4489548	0.039215688	0.20438658
WELCSH_BRCA1_TARGETS_UP	191	1.4488534	0.036538463	0.2041474
HESS_TARGETS_OF_HOXA9_AND_MEIS1_DN	76	1.4488207	0.04158416	0.20379569
AKL_HTLV1_INFECTION_UP	25	1.4482987	0.017681729	0.20442164
GARGALOVIC_RESPONSE_TO_OXIDIZED_PHOSPHOLIPIDS_BLACK_UP	34	1.4479564	0.03968254	0.20428368
WU_HBX_TARGETS_3_UP	18	1.4477054	0.038240917	0.20430772
VARELA_ZMPSTE24_TARGETS_UP	39	1.4475158	0.027184466	0.20340799
GENTILE_UV_RESPONSE_CLUSTER_D2	40	1.4474478	0.048076924	0.20315273
KYNG_DNA_DAMAGE_BY_GAMMA_RADIATION	78	1.4472003	0.038854804	0.20240137
CAFFAREL_RESPONSE_TO_THC_24HR_5_DN	56	1.4469845	0.049115915	0.2024113
DEMAGALHAES_AGING_UP	55	1.4468279	0.029239766	0.20189594
WALLACE_PROSTATE_CANCER_RACE_UP	277	1.4463493	0.04637097	0.20208217
FOURNIER_ACINAR_DEVELOPMENT_LATE_2	263	1.4461242	0.04483431	0.20215346
KIM_WT1_TARGETS_12HR_DN	195	1.4458019	0.017142856	0.2023901
ZHAN_LATE_DIFFERENTIATION_GENES_UP	32	1.4453802	0.031496063	0.20200361
FARMER_BREAST_CANCER_APOCRINE_VS_BASAL	319	1.4451637	0.015533981	0.20213637
PID_RETINOIC_ACID_PATHWAY	29	1.4451462	0.035019454	0.20175757
SASSON_RESPONSE_TO_GONADOTROPHINS_UP	87	1.4449071	0.025145067	0.20176932
SHAFFER_IRF4_TARGETS_IN_MYELOMA_VS_MATURE_B_LYMPHOCYTE	99	1.4444689	0.035363458	0.20221493
NELSON_RESPONSE_TO_ANDROGEN_UP	85	1.4442828	0.032882012	0.20177758
CASORELLI_ACUTE_PROMYELOCYTIC_LEUKEMIA_UP	164	1.4442427	0.017821781	0.20146737
ELVIDGE_HIF1A_AND_HIF2A_TARGETS_UP	38	1.4439226	0.0407767	0.20125072
SHEPARD_BMYB_MORPHOLINO_UP	197	1.443773	0.033333335	0.2011494
ROSS_AML_WITH_CBFB_MYH11_FUSION	50	1.4437128	0.033203125	0.20086503
RUTELLA_RESPONSE_TO_HGF_VS_CSF2RB_AND_IL4_UP	393	1.4430394	0.03952569	0.2018507
CHENG_RESPONSE_TO_NICKEL_ACETATE	41	1.4424179	0.040384617	0.20256771
FERRANDO_LYL1_NEIGHBORS	15	1.4421353	0.041257367	0.20235863
FARMER_BREAST_CANCER_APOCRINE_VS_LUMINAL	309	1.442035	0.02	0.20134556

**BROWN_MYELOID_CELL_DEVELOPMENT_UP**	157	1.4419136	0.0390625	0.20117326
**REACTOME_SPHINGOLIPID_DE_NOVO_BIOSYNTHESIS**	25	1.4416708	0.025	0.20127709
**BRACHAT_RESPONSE_TO_METHOTREXATE_DN**	25	1.4416382	0.033203125	0.20096435
**BIOCARTA_CHREBP2_PATHWAY**	42	1.4416214	0.026871402	0.20061138
**LINDSTEDT_DENDRITIC_CELL_MATURATION_D**	68	1.4414831	0.024761904	0.20049341
**PENG_GLUTAMINE_DEPRIVATION_UP**	38	1.4411407	0.043222003	0.20073918
**REACTOME_ACTIVATION_OF_THE_PRE_REPLICATIVE_COMPLEX**	24	1.4399216	0.04950495	0.2008437
**VANTVEER_BREAST_CANCER_METASTASIS_DN**	111	1.4396454	0.038383838	0.19955207
**HIRSCH_CELLULAR_TRANSFORMATION_SIGNATURE_UP**	236	1.4396262	0.015564202	0.19922654
**RODWELL_AGING_KIDNEY_UP**	443	1.4396198	0.023483366	0.19886665
**ST_TUMOR_NECROSIS_FACTOR_PATHWAY**	28	1.4395857	0.040697675	0.19855791
**REACTOME_INTEGRATION_OF_ENERGY_METABOLISM**	118	1.4393116	0.02734375	0.19863829
**HOLLMANN_APOPTOSIS_VIA_CD40_UP**	190	1.439101	0.045454547	0.19868264
**ZUCCHI_METASTASIS_DN**	44	1.4389151	0.048543688	0.19865057
**KYNG_DNA_DAMAGE_DN**	192	1.4388145	0.041420117	0.19847967
**FUJII_YBX1_TARGETS_UP**	42	1.4385655	0.03877551	0.19859533
**HUMMERICH_SKIN_CANCER_PROGRESSION_UP**	88	1.4385365	0.033398822	0.1978999
**WANG_CISPLATIN_RESPONSE_AND_XPC_DN**	218	1.4384984	0.011952192	0.19759431
**GOTZMANN_EPITHELIAL_TO_MESENCHYMAL_TRANSITION_DN**	203	1.4381257	0.04950495	0.19801383
**BRACHAT_RESPONSE_TO_CAMPTOTHECIN_DN**	41	1.4367781	0.032882012	0.19957037
**THUM_SYSTOLIC_HEART_FAILURE_UP**	393	1.4365736	0.034	0.19956228
**GENTILE_UV_RESPONSE_CLUSTER_D1**	18	1.4362411	0.048582997	0.19975875
**BERENJENO_ROCK_SIGNALING_NOT_VIA_RHOA_UP**	29	1.4359007	0.03529412	0.19934379
**ZAMORA_NOS2_TARGETS_DN**	94	1.4346354	0.036821704	0.20057382
**GRUETZMANN_PANCREATIC_CANCER_UP**	351	1.4343752	0.04133858	0.20079619
**HOSHIDA_LIVER_CANCER_SUBCLASS_S1**	232	1.4340945	0.021568628	0.20070386
**KYNG_ENVIRONMENTAL_STRESS_RESPONSE_NOT_BY_UV_IN_OLD**	24	1.4340758	0.048732944	0.20037952
**MARIADASON_RESPONSE_TO_BUTYRATE_SULINDAC_4**	20	1.4337964	0.031189084	0.19987436
**HOFMANN_CELL_LYMPHOMA_UP**	50	1.4334409	0.03187251	0.20000558
**CHANDRAN_METASTASIS_TOP50_DN**	44	1.4323373	0.022988506	0.20117053
**SAMOLS_TARGETS_OF_KHSV_MIRNAS_DN**	57	1.4319996	0.027079303	0.20142905
**HOEBEKE_LYMPHOID_STEM_CELL_DN**	83	1.4319347	0.04509804	0.20119178
**SHIN_B_CELL_LYMPHOMA_CLUSTER_9**	18	1.431624	0.016563147	0.2003746
**LI_INDUCED_T_TO_NATURAL_KILLER_UP**	290	1.4315621	0.035019454	0.20015976
**LIU_SOX4_TARGETS_UP**	131	1.4309552	0.033333335	0.20066133
**THEILGAARD_NEUTROPHIL_AT_SKIN_WOUND_DN**	219	1.4303721	0.049212597	0.20074165
**AKL_HTLV1_INFECTION_DN**	64	1.4300462	0.029821074	0.20071188
**OSWALD_HEMATOPOIETIC_STEM_CELL_IN_COLLAGEN_GEL_UP**	225	1.4299767	0.015936255	0.20050228
**TONG_INTERACT_WITH_PTTG1**	52	1.4291956	0.03307393	0.20020553
**SEKI_INFLAMMATORY_RESPONSE_LPS_UP**	74	1.4289377	0.04183267	0.20034075
**FERRANDO_T_ALL_WITH_MLL_ENL_FUSION_UP**	86	1.4285892	0.028355388	0.20067781

GOLDRATH_ANTIGEN_RESPONSE	335	1.4284855	0.049115915	0.20055318
PEDERSEN_METASTASIS_BY_ERBB2_ISOFORM_4	105	1.4276938	0.02173913	0.20108652
PID_ERBB1_DOWNSTREAM_PATHWAY	103	1.4273788	0.03550296	0.20064856
RAY_TARGETS_OF_P210_BCR_ABL_FUSION_DN	16	1.4270401	0.04950495	0.2006602
LINDSTEDT_DENDRITIC_CELL_MATURATION_C	68	1.4267913	0.035363458	0.20086169
KAUFFMANN_DNA_REPLICATION_GENES	137	1.4267441	0.04606526	0.20062631
BERTUCCI_MEDULLARY_VS_DUCTAL_BREAST_CANCER_UP	191	1.4263692	0.0390625	0.2010609
HOLLMANN_APOPTOSIS_VIA_CD40_DN	250	1.4263337	0.03522505	0.20078944
RUTELLA_RESPONSE_TO_CSF2RB_AND_IL4_UP	326	1.4259839	0.031746034	0.20084253
SEITZ_NEOPLASTIC_TRANSFORMATION_BY_8P_DELETION_DN	30	1.425102	0.04133858	0.20160203
WANG_RECURRENT_LIVER_CANCER_DN	16	1.4250776	0.04863813	0.20131202
COATES_MACROPHAGE_M1_VS_M2_DN	70	1.4247619	0.041749503	0.20151909
LIU_SOX4_TARGETS_DN	291	1.4246192	0.034951456	0.20143603
WALLACE_JAK2_TARGETS_UP	24	1.4244909	0.049523808	0.20130771
REACTOME_REGULATION_OF_KIT_SIGNALING	17	1.4239879	0.04191617	0.2003151
LABBE_TARGETS_OF_TGFB1_AND_WNT3A_UP	109	1.4230118	0.044573642	0.19989794
APRELIKOVA_BRCA1_TARGETS	49	1.4226924	0.03696498	0.19949424
MCBRYAN_PUBERTAL_BREAST_4_5WK_DN	185	1.4224294	0.02016129	0.19929878
REACTOME_MAPK_TARGETS_NUCLEAR_EVENTS_MEDIATED_BY_MAP_KINASES	30	1.4223742	0.041904762	0.19908077
SHETH_LIVER_CANCER_VS_TXNIP_LOSS_PAM1	221	1.4219369	0.023210831	0.1984217
SHEPARD_CRUSH_AND_BURN_MUTANT_UP	186	1.4217001	0.032818533	0.19790219
BURTON_ADIPOGENESIS_12	30	1.4214846	0.041420117	0.19796763
JIANG_HYPOXIA_CANCER	78	1.4214509	0.04606526	0.19773677
REACTOME_TRANSCRIPTIONAL_REGULATION_OF_WHITE_ADIPOCYTE_DIFFERENTIATION	70	1.4205791	0.04109589	0.19819407
BYSTRYKH_HEMATOPOIESIS_STEM_CELL_AND_BRAIN_QTL_TRANS	175	1.4204888	0.03646833	0.19744013
JOHNSTONE_PARVB_TARGETS_2_UP	135	1.4203668	0.020833334	0.19735296
AGUIRRE_PANCREATIC_CANCER_COPY_NUMBER_DN	217	1.4193898	0.04597701	0.19844371
WOOD_EBV_EBNA1_TARGETS_UP	110	1.4193314	0.044487428	0.19824389
GARGALOVIC_RESPONSE_TO_OXIDIZED_PHOSPHOLIPIDS_GREEN_UP	23	1.4188923	0.041825093	0.19780645
LUI_THYROID_CANCER_CLUSTER_1	49	1.41838	0.024	0.19751209
RIGGINS_TAMOXIFEN_RESISTANCE_DN	214	1.4180951	0.027613413	0.1977499
PID_TRAIL_PATHWAY	28	1.4175515	0.03952569	0.19781253
KEGG_CHEMOKINE_SIGNALING_PATHWAY	188	1.417496	0.015873017	0.19759427
HOFFMANN_SMALL_PRE_BII_TO_IMMATURE_B_LYMPHOCYTE_DN	50	1.4174112	0.023483366	0.19744635
SESTO_RESPONSE_TO_UV_C5	46	1.417382	0.048828125	0.19719689
REACTOME_G1_PHASE	37	1.4165213	0.04255319	0.19677778
WEIGEL_OXIDATIVE_STRESS_BY_TBH_AND_H2O2	35	1.4148192	0.03929273	0.19754297
BIOCARTA_MET_PATHWAY	37	1.4146954	0.03353057	0.1974791
GROSS_HYPOXIA_VIA_ELK3_DN	150	1.4146127	0.04330709	0.19732976
REACTOME_MHC_CLASS_II_ANTIGEN_PRESENTATION	91	1.4145036	0.033333335	0.1971607
MAHAJAN_RESPONSE_TO_IL1A_UP	81	1.4122285	0.042462844	0.1990607

LEIN_OLIGODENDROCYTE_MARKERS	69	1.4122274	0.038986355	0.1987765
KEGG_NOD_LIKE_RECEPTOR_SIGNALING_PATHWAY	59	1.4114577	0.03937008	0.19960748
PID_ILK_PATHWAY	45	1.4080392	0.027504912	0.1998721
GAL_LEUKEMIC_STEM_CELL_DN	231	1.4069612	0.033009708	0.19990104
ZHONG_SECRETOME_OF_LUNG_CANCER_AND_ENDOTHELIUM	65	1.4068204	0.045454547	0.19955413
UEDA_PERIFERAL_CLOCK	159	1.405421	0.031496063	0.19868033
NIELSEN_LEIOMYOSARCOMA_UP	17	1.4051892	0.026209677	0.19779873
ZHANG_BREAST_CANCER_PROGENITORS_DN	140	1.4042321	0.0407767	0.19634518
PID_TNFPATHWAY	44	1.4034581	0.033009708	0.196922
BILD_HRAS_ONCOGENIC_SIGNATURE	248	1.4033405	0.032520324	0.19688185
REACTOME_MAP_KINASE_ACTIVATION_IN_TLR_CASCADE	46	1.402121	0.04054054	0.1962722
MAGRANGEAS_MULTIPLE_MYELOMA_IGLL_VS_IGLK_UP	40	1.4018964	0.021359224	0.19587065
WHITFIELD_CELL_CYCLE_G2	165	1.3993015	0.03353057	0.19697462
JAATINEN_HEMATOPOIETIC_STEM_CELL_DN	213	1.39919	0.041420117	0.19688626
NOUZOVA_METHYLATED_IN_APL	58	1.398552	0.028409092	0.19746159
KEEN_RESPONSE_TO_ROSIGLITAZONE_DN	104	1.3963083	0.04158416	0.1981765
OUYANG_PROSTATE_CANCER_PROGRESSION_UP	19	1.3951851	0.04527559	0.19938174
REACTOME_NUCLEAR_EVENTS_KINASE_AND_TRANSCRIPTION_FACTOR_ACTIVATION	24	1.3950818	0.047709923	0.19928803
WIERENGA_STAT5A_TARGETS_UP	204	1.3950083	0.03515625	0.19892493
IGLESIAS_E2F_TARGETS_UP	150	1.3938861	0.04901961	0.19956821
LUI_THYROID_CANCER_PAX8_PPARG_UP	43	1.3927048	0.03245436	0.20002839
YORDY_RECIPROCAL_REGULATION_BY_ETS1_AND_SP100_UP	21	1.3924973	0.037523452	0.20007755
CADWELL_ATG16L1_TARGETS_DN	60	1.392442	0.023121387	0.19991656
RICKMAN_TUMOR_DIFFERENTIATED_MODERATELY_VS_POORLY_UP	112	1.3922454	0.039447732	0.19974175
LEE_LIVER_CANCER_DENA_UP	59	1.3920181	0.04892368	0.1996334
DELASERNA_MYOD_TARGETS_DN	55	1.3898811	0.03929273	0.20043284
IWANAGA_CARCINOGENESIS_BY_KRAS_UP	154	1.3848802	0.05	0.20179583
LASTOWSKA_COAMPLIFIED_WITH_MYCN	40	1.3847269	0.039033458	0.20135136
LIM_MAMMARY_STEM_CELL_DN	404	1.3836287	0.04296875	0.20130512
JIANG_HYPOXIA_NORMAL	296	1.3829882	0.04263566	0.20116593
BANDRES_RESPONSE_TO_CARMUSTIN_MG	159	1.3821402	0.03256705	0.20184582
KEGG_AMINO_SUGAR_AND_NUCLEOTIDE_SUGAR_METABOLISM	44	1.3819085	0.04423077	0.20206337
HAN_SATB1_TARGETS_DN	421	1.3806916	0.041501977	0.2020063
YANG_BCL3_TARGETS_UP	347	1.375523	0.030991735	0.20453852
KEGG_CYTOKINE_CYTOKINE_RECEPTOR_INTERACTION	265	1.370787	0.046709128	0.20557475
ZHU_CMV_8_HR_DN	53	1.3706459	0.042424243	0.20538308
FARMER_BREAST_CANCER_BASAL_VS_LULMINAL	315	1.3697442	0.036190476	0.205419
YAGI_AML_RELAPSE_PROGNOSIS	35	1.3693016	0.04536862	0.20547073
SANSOM_APC_TARGETS_REQUIRE_MYC	196	1.3692548	0.04296875	0.20533429
WANG_ESOPHAGUS_CANCER_VS_NORMAL_UP	116	1.3672936	0.027777778	0.20669007
NAKAMURA_ADIPOGENESIS_LATE_UP	99	1.3586439	0.041501977	0.20981163
GREGORY_SYNTHETIC_LETHAL_WITH_IMATINIB	139	1.3500751	0.031189084	0.21257612

**HELLER_SILENCED_BY_METHYLATION_UP**	277	1.3488044	0.04255319	0.21298103
**ACEVEDO_FGFR1_TARGETS_IN_PROSTATE_CANCER_MODEL_UP**	280	1.3425481	0.033663366	0.21520826

Table I.III The top 13 C2 gene sets most highly enriched in macrophages from symptomatic plaques. Statistical threshold set as P < 0.01, FDR Q < 0.25. NES = normalized enrichment score.

Pathway name	Size in gene set	NES	P-val	FDR Q-val
**WENG_POR_TARGETS_LIVER_UP**	38	1.5114162	0.003752345	0.24482584
**GNATENKO_PLATELET_SIGNATURE**	46	1.5066727	0.005758158	0.24098971
**RASHI_RESPONSE_TO_IONIZING_RADIATION_6**	79	1.5040202	0.007843138	0.24226007
**MELLMAN_TUT1_TARGETS_DN**	47	1.5025938	0.007858546	0.24104062
**AMUNDSON_GENOTOXIC_SIGNATURE**	101	1.5013996	0.007692308	0.24125974
**BRUNO_HEMATOPOIESIS**	64	1.4996777	0.003976143	0.24084347
**MOOTHA_TCA**	16	1.4961965	0.007707129	0.24340804
**REACTOME_TRAF6_MEDIATED_INDUCTION_OF_NFKB_AND_MAP_KINASES_UPON_TLR7_8_OR_9_ACTIVATION**	72	1.4960093	0.007797271	0.24196623
**GAZDA_DIAMOND_BLACKFAN_ANEMIA_PROGENITOR_UP**	39	1.4937316	0.007751938	0.23292744
**NIKOLSKY_OVERCONNECTED_IN_BREAST_CANCER**	22	1.49074	0.008	0.22668043
**GROSS_HYPOXIA_VIA_ELK3_AND_HIF1A_UP**	136	1.4681828	0.007984032	0.20276564
**KYNG_RESPONSE_TO_H2O2**	71	1.4672376	0.007490637	0.20268361
**YAGI_AML_SURVIVAL**	122	1.4558709	0.009652509	0.19807208

Table I.IV Only 1 C2 gene set was significantly enriched in macrophages from asymptomatic plaques with P < 0.01 and FDR Q < 0.25. NES = normalized enrichment score.

Pathway name	Size in gene set	NES	P-val	FDR Q-val
**REACTOME_TERMINATION_OF_O_GLYCAN_BIOSYNTHESIS**	24	1.884635	< 0.001	0.014480328

Table I.V 93 C5 gene sets were significantly enriched in macrophages from symptomatic plaques with P < 0.05 and FDR Q < 0.25. NES = normalized enrichment score.

Pathway name	Size in gene set	NES	P-val	FDR Q-val
**RHYTHMIC_PROCESS**	29	1.7336898	0.001879699	0.23356893
**REGULATION_OF_I_KAPPAB_KINASE_NF_KAPPAB_CASCADE**	90	1.5644419	0.001923077	0.2471346
**POSITIVE_REGULATION_OF_I_KAPPAB_KINASE_NF_KAPPAB_CASCADE**	84	1.5519826	0.001937985	0.2387998
**INFLAMMATORY_RESPONSE**	127	1.5089182	0.007633588	0.21578052
**I_KAPPAB_KINASE_NF_KAPPAB_CASCADE**	111	1.511637	0.007648184	0.22150522
**LIPID_RAFT**	29	1.5018569	0.007766991	0.20646443
**PHAGOCYTOSIS**	17	1.6794552	0.009451796	0.2085065
**DEFENSE_RESPONSE**	267	1.4956425	0.00967118	0.20747738
**RIBOSOMAL_SUBUNIT**	20	1.5532049	0.0113852	0.24673446
**CALCIUM_ION_BINDING**	101	1.4926146	0.011472276	0.19942274
**REGULATION_OF_PROTEIN_STABILITY**	19	1.5774672	0.011764706	0.21050487
**MITOCHONDRIAL_RIBOSOME**	22	1.550192	0.013645224	0.22147482
**ORGANELLAR_RIBOSOME**	22	1.5501921	0.013645224	0.2333813
**POSITIVE_REGULATION_OF_SIGNAL_TRANSDUCTION**	123	1.5024319	0.015037594	0.21733322
**DNA_DIRECTED_RNA_POLYMERASEII_HOLOENZYME**	65	1.5214517	0.015444015	0.22062426
**IMMUNE_RESPONSE**	234	1.5285498	0.015473888	0.21263608
**CHEMOKINE_RECEPTOR_BINDING**	43	1.5600088	0.015655577	0.2345546
**PERINUCLEAR_REGION_OF_CYTOPLASM**	54	1.5018796	0.01663586	0.21267992
**POSITIVE_REGULATION_OF_PHOSPHATE_METABOLIC_PROCESS**	28	1.5379771	0.017175572	0.20483726
**SMALL_NUCLEAR_RIBONUCLEOPROTEIN_COMPLEX**	22	1.5711383	0.017274473	0.24736058
**NUCLEAR_CHROMOSOME**	53	1.4906088	0.017408123	0.2001
**POSITIVE_REGULATION_OF_PHOSPHORYLATION**	26	1.5709763	0.017408123	0.23175457
**IMMUNE_EFFECTOR_PROCESS**	38	1.575469	0.017578125	0.24784433
**CHEMOKINE_ACTIVITY**	42	1.5422351	0.019417476	0.21814152
**INTRINSIC_TO_ORGANELLE_MEMBRANE**	51	1.467603	0.020560747	0.1894704
**LIPOPROTEIN_BIOSYNTHETIC_PROCESS**	26	1.4947524	0.020637898	0.20418488
**RESPONSE_TO_ABIOTIC_STIMULUS**	89	1.5281713	0.021568628	0.20593684
**EARLY_ENDOSOME**	18	1.4332556	0.021653544	0.17127567
**SMALL_PROTEIN_CONJUGATING_ENZYME_ACTIVITY**	52	1.395784	0.021912351	0.1629684
**INTEGRAL_TO_ORGANELLE_MEMBRANE**	49	1.4579377	0.02238806	0.1862281
**COATED_MEMBRANE**	16	1.516247	0.02264151	0.21503978
**MEMBRANE_COAT**	16	1.516247	0.02264151	0.2227237
**SMALL_CONJUGATING_PROTEIN_LIGASE_ACTIVITY**	51	1.3726761	0.02414487	0.16640734
**CELLULAR_PROTEIN_COMPLEX_ASSEMBLY**	33	1.4505298	0.02414487	0.17391174
**PROTEIN_AMINO_ACID_LIPIDATION**	24	1.4579791	0.024621213	0.1897057
**COATED_VESICLE_MEMBRANE**	16	1.4733237	0.024809161	0.18957775

**MEDIATOR_COMPLEX**	18	1.5177045	0.024952015	0.22571292
**CHROMATIN**	35	1.4875156	0.025242718	0.19431665
**TRANS_GOLGI_NETWORK**	20	1.436507	0.025878003	0.17272493
**MAINTENANCE_OF_LOCALIZATION**	21	1.4711906	0.025896415	0.1835008
**CLATHRIN_COATED_VESICLE**	36	1.448904	0.026515152	0.16934537
**VESICLE_COAT**	15	1.4859787	0.026819924	0.1892176
**ACID_AMINO_ACID_LIGASE_ACTIVITY**	57	1.3899032	0.03	0.15724304
**GOLGI_MEMBRANE**	44	1.4613415	0.030018762	0.19558158
**RESPONSE_TO_ORGANIC_SUBSTANCE**	30	1.4749484	0.030534351	0.19275542
**CYTOKINE_ACTIVITY**	111	1.4773937	0.03065134	0.190625
**TRANSLATIONAL_INITIATION**	39	1.46564	0.03065134	0.19116613
**CELLULAR_BIOSYNTHETIC_PROCESS**	317	1.4595987	0.03065134	0.19641297
**ADAPTIVE_IMMUNE_RESPONSE**	25	1.5344459	0.031189084	0.19940435
**RECEPTOR_SIGNALING_PROTEIN_ACTIVITY**	81	1.4032328	0.0332681	0.16640096
**ER_TO_GOLGI_VESICLE_MEDIATED_TRANSPORT**	18	1.4776131	0.03358209	0.194585
**IMMUNE_SYSTEM_PROCESS**	327	1.4182749	0.034416825	0.16201752
**REGULATION_OF_PROTEIN_MODIFICATION_PROCESS**	44	1.4589574	0.035714287	0.19086244
**TRANSPORT_VESICLE**	30	1.4561682	0.03731343	0.18786877
**TRANSLATION**	178	1.4886987	0.03816794	0.19596236
**UBIQUITIN_PROTEIN_LIGASE_ACTIVITY**	49	1.3312191	0.038383838	0.1602959
**MITOCHONDRION**	333	1.5414728	0.03846154	0.21106982
**RNA_PROCESSING**	153	1.5011083	0.038535647	0.2024962
**MITOCHONDRIAL_PART**	140	1.5603333	0.03883495	0.24798187
**RESPONSE_TO_OXIDATIVE_STRESS**	45	1.5479625	0.038986355	0.21894553
**NUCLEOBASENUCLEOSIDENUCLEOTIDE_AND_NUCLEIC_ACID_TRANSPORT**	30	1.4068563	0.03952569	0.16930514
**PHOSPHOLIPID_BIOSYNTHETIC_PROCESS**	39	1.4223614	0.039772727	0.17214998
**POSITIVE_REGULATION_OF_PROTEIN_MODIFICATION_PROCESS**	29	1.4217628	0.04	0.17103093
**INTRACELLULAR_TRANSPORT**	274	1.41783	0.040076334	0.16088688
**GTP_BINDING**	46	1.5077931	0.040618956	0.21254557
**LIPOPROTEIN_METABOLIC_PROCESS**	33	1.4560287	0.040892195	0.18500804
**RESPONSE_TO_UV**	26	1.4379443	0.040983606	0.17201164
**BIOSYNTHETIC_PROCESS**	464	1.421279	0.041509435	0.17038226
**LOCOMOTORY_BEHAVIOR**	92	1.3671521	0.04158416	0.1645499
**DNA_POLYMERASE_ACTIVITY**	18	1.4870712	0.041666668	0.19070956
**MITOCHONDRIAL_MEMBRANE**	84	1.537514	0.042471044	0.1974373
**TRANSFERASE_ACTIVITY_TRANSFERRING_GROUPS_OTHER_THAN_AMINO_ACYL_GROUPS**	41	1.4264069	0.042718448	0.16510423
**GUANYL_NUCLEOTIDE_BINDING**	47	1.4984096	0.042718448	0.20497107
**UNFOLDED_PROTEIN_BINDING**	41	1.4819047	0.043071162	0.19134813
**GOLGI_APPARATUS_PART**	98	1.4299817	0.04347826	0.16833716
**G_PROTEIN_COUPLED_RECEPTOR_BINDING**	54	1.4171944	0.04389313	0.16035523
**NUCLEAR_EXPORT**	32	1.4494292	0.044145875	0.17075174
**ENDOSOME**	66	1.3931307	0.044660196	0.1570796
**ENDOSOME_TRANSPORT**	23	1.3898485	0.045009784	0.15580144

**CELLULAR_CATABOLIC_PROCESS**	212	1.4436873	0.045009784	0.17197665
**PHOSPHOINOSITIDE_BIOSYNTHETIC_PROCESS**	24	1.3986485	0.045801528	0.1602161
**REGULATION_OF_TRANSLATIONAL_INITIATION**	31	1.4389036	0.045889102	0.1724432
**ANTIGEN_BINDING**	22	1.4932477	0.046692606	0.2031714
**FATTY_ACID_OXIDATION**	18	1.4798508	0.04725898	0.1929277
**VESICLE_MEDIATED_TRANSPORT**	193	1.4095914	0.04761905	0.164835
**MITOCHONDRIAL_INNER_MEMBRANE**	65	1.5450463	0.04780115	0.21767493
**RIBONUCLEOPROTEIN_COMPLEX_BIOGENESIS_AND_ASSEMBLY**	76	1.4429879	0.047984645	0.17072504
**CATABOLIC_PROCESS**	224	1.4311506	0.048732944	0.16816073
**DOUBLE_STRANDED_RNA_BINDING**	17	1.4499286	0.048828125	0.1724236
**MACROMOLECULE_BIOSYNTHETIC_PROCESS**	319	1.4122614	0.04887218	0.16295003
**ENDOPLASMIC_RETICULUM_MEMBRANE**	84	1.455566	0.048964217	0.1792344
**INTRINSIC_TO_GOLGI_MEMBRANE**	15	1.4012576	0.049701788	0.1631853
**COFACTOR_METABOLIC_PROCESS**	53	1.5407996	0.04990403	0.20388042

Table II.I 72 significantly enriched C2 pathways in macrophages (core and cap) from carotid plaques containing large lipid content (>25% Lipid Area) as detected by MRI T_2 mapping. Threshold P < 0.05, FDR < 0.25. NES = normalized enrichment score

Pathway name	Size in gene set	NES	P-val	FDR Q-val
EINAV_INTERFERON_SIGNATURE_IN_CANCER	27	1.5974699	0.022633744	0.24895108
REACTOME_ANTIGEN_PROCESSING_CROSS_PRESENTATION	74	1.5732976	0.016129032	0.24769165
REACTOME_ER_PHAGOSOME_PATHWAY	59	1.5717558	0.016194332	0.24965818
TAVOR_CEBPA_TARGETS_UP	47	1.5717279	0.004065041	0.24687399
REACTOME_P53_DEPENDENT_G1_DNA_DAMAGE_RESPONSE	55	1.5709977	0.024	0.2463786
HAHTOLA_CTCL_CUTANEOUS	26	1.5705765	0.018442623	0.24506004
CAFFAREL_RESPONSE_TO_THC_24HR_5_UP	31	1.5540613	0.011904762	0.24928048
BENPORATH_PROLIFERATION	137	1.553627	0.044145875	0.24844381
KEGG_PORPHYRIN_AND_CHLOROPHYLL_METABOLISM	41	1.5528826	0.024640657	0.24814849
PRAMOONJAGO_SOX4_TARGETS_UP	49	1.5527544	0.022357723	0.2463354
PID_IFNGPATHWAY	39	1.5519499	0.004081633	0.24644986
JACKSON_DNMT1_TARGETS_UP	77	1.5517613	0.022	0.24478626
ALCALA_APOPTOSIS	86	1.5514604	0.018404908	0.24355878
REACTOME_CDK_MEDIATED_PHOSPHORYLATION_AND_REMOVAL_OF_CDC6	47	1.5512561	0.030120483	0.24198502
REACTOME_CELL_CYCLE_CHECKPOINTS	112	1.5490972	0.03187251	0.24446274
FULCHER_INFLAMMATORY_RESPONSE_LECTIN_VS_LPS_DN	430	1.548959	0.008163265	0.24282087
CHANG_IMMORTALIZED_BY_HPV31_DN	64	1.5477376	0.013435701	0.24470448
LENAOUR_DENDRITIC_CELL_MATURATION_DN	127	1.5461832	0.013916501	0.24752015
PELLICCIOTTA_HDAC_IN_ANTIGEN_PRESENTATION_DN	49	1.5458522	0.03629032	0.24645306
REACTOME_P53_INDEPENDENT_G1_S_DNA_DAMAGE_CHECKPOINT	50	1.5455699	0.020491803	0.24527808
CHUNG_BLISTER_CYTOTOXICITY_UP	126	1.545433	0.022088353	0.24368535
ZHU_CMV_24_HR_UP	87	1.5448447	0.012121212	0.24345483
REACTOME_CYTOKINE_SIGNALING_IN_IMMUNE_SYSTEM	264	1.5445395	0.014084507	0.24254717
KEGG_TOLL_LIKE_RECEPTOR_SIGNALING_PATHWAY	100	1.5444627	0.020408163	0.24088882
XU_AKT1_TARGETS_6HR	26	1.5440959	0.031120332	0.24004014
REACTOME_REGULATION_OF_IFNA_SIGNALING	24	1.542886	0.044989776	0.23990346
GOLUB_ALL_VS_AML_DN	24	1.5418373	0.023762377	0.24112871
RUTELLA_RESPONSE_TO_HGF_VS_CSF2RB_AND_IL4_UP	393	1.5417224	0.02745098	0.23973913
VILIMAS_NOTCH1_TARGETS_DN	20	1.5396484	0.024242423	0.24396884
TARTE_PLASMA_CELL_VS_PLASMABLAST_DN	302	1.539056	0.03984064	0.24409102
VERHAAK_AML_WITH_NPM1_MUTATED_UP	180	1.5389129	0.014285714	0.24257474
KEGG_PROTEASOME	46	1.5386915	0.038617887	0.24146692
NOJIMA_SFRP2_TARGETS_DN	25	1.5384821	0.011976048	0.2401832

REACTOME_CITRIC_ACID_CYCLE_TCA_CYCLE	23	1.5381352	0.020876827	0.23947226
LIAN_LIPA_TARGETS_6M	72	1.537518	0.012244898	0.23943807
BIOCARTA_MPR_PATHWAY	33	1.5356623	0.010162601	0.24276935
REACTOME_GLUCOSE_TRANSPORT	38	1.535104	0.038306452	0.24261639
REACTOME_AUTODEGRADATION_OF_THE_E3_UBIQUITIN_LIGASE_COP1	49	1.5345644	0.022494888	0.24240388
DAZARD_RESPONSE_TO_UV_SCC_UP	114	1.534419	0.0332681	0.24121417
ZHU_CMV_ALL_UP	114	1.5342325	0.018108651	0.24002223
PAPASPYRIDONOS_UNSTABLE_ATEROSCLEROTIC_PLAQUE_UP	50	1.5341467	0.037623763	0.23866367
KEGG_NITROGEN_METABOLISM	23	1.5327405	0.03869654	0.24065809
PID_TOLL_ENDOGENOUS_PATHWAY	25	1.5318885	0.01443299	0.24166556
BIOCARTA_CDC42RAC_PATHWAY	16	1.5317639	0.021912351	0.24032849
BOYAULT_LIVER_CANCER_SUBCLASS_G3_UP	184	1.5315595	0.045908183	0.23925987
MOOTHA_HUMAN_MITODB_6_2002	418	1.5311809	0.044088177	0.23857982
RUAN_RESPONSE_TO_TROGLITAZONE_UP	24	1.5310782	0.032520324	0.23723798
BIOCARTA_41BB_PATHWAY	17	1.530749	0.031055901	0.23647171
SHIPP_DLBCL_VS_FOLLICULAR_LYMPHOMA_UP	45	1.5297024	0.03006012	0.2372898
KEGG_CITRATE_CYCLE_TCA_CYCLE	32	1.5290573	0.024439918	0.23798706
LY_AGING_OLD_DN	55	1.5287704	0.035643563	0.23721232
SHARMA_PILOCYTIC_ASTROCYTOMA_LOCATION_UP	24	1.5282121	0.01622718	0.23721
YAO_TEMPORAL_RESPONSE_TO_PROGESTERONE_CLUSTER_13	160	1.5256032	0.048387095	0.24064209
REACTOME_DNA_REPLICATION	183	1.5252736	0.04901961	0.23997411
REACTOME_CROSS_PRESENTATION_OF_SOLUBLE_EXOGENOUS_ANTIGENS_ENDOSOMES	48	1.524808	0.04819277	0.23686178
REACTOME_APC_C_CDC20_MEDIATED_DEGRADATION_OF_CYCLIN_B	22	1.523689	0.04347826	0.23851733
GARGALOVIC_RESPONSE_TO_OXIDIZED_PHOSPHOLIPIDS_YELLOW_UP	26	1.5234045	0.021912351	0.23777136
REACTOME_DESTABILIZATION_OF_MRNA_BY_BRF1	17	1.5217061	0.018329939	0.24077147
SESTO_RESPONSE_TO_UV_C3	20	1.5208337	0.037037037	0.24182242
PARK_HSC_VS_MULTIPOTENT_PROGENITORS_DN	17	1.5202718	0.033464566	0.24185173
BIOCARTA_TID_PATHWAY	19	1.5200062	0.030991735	0.24112174
REACTOME_DESTABILIZATION_OF_MRNA_BY_AUF1_HNRNP_D0	52	1.5198694	0.03846154	0.23999318
BRACHAT_RESPONSE_TO_METHOTREXATE_DN	25	1.5179381	0.00610998	0.24394432
AMIT_SERUM_RESPONSE_20_MCF10A	21	1.5176522	0.029126214	0.24329337
KOINUMA_COLON_CANCER_MSI_DN	16	1.5167948	0.034	0.24429756
HU_ANGIOGENESIS_DN	37	1.5165755	0.045816734	0.24344231
BIOCARTA_ACTINY_PATHWAY	20	1.5157374	0.04040404	0.24418472
JISON_SICKLE_CELL_DISEASE_UP	178	1.5156785	0.028	0.24295089
CHOW_RASSF1_TARGETS_DN	29	1.5148889	0.022540983	0.24364522
BIOCARTA_TOLL_PATHWAY	35	1.5145446	0.01632653	0.24314828
REACTOME_REGULATION_OF_APOPTOSIS	57	1.5142158	0.049382716	0.24273741
REACTOME_REGULATION_OF_GLUCOKINASE_BY_GLUCOKINASE_REGULATORY_PROTEIN	27	1.5120791	0.042857144	0.24728873

Table II.II 5 significantly enriched C2 pathways in macrophages (core only) from carotid plaques computed using Lipid Area (%) directly measured by MRI T2 mapping, as a *continuous variable,* for GSEA. Threshold P < 0.05, FDR < 0.25. NES = normalized enrichment score

Pathway name	Size in gene set	NES	P-val	FDR Q-val
**MOSERLE_IFNA_RESPONSE**	31	1.8810172	0.005940594	0.17646831
**ZHANG_INTERFERON_RESPONSE**	23	1.8438345	0.01010101	0.2453157
**HECKER_IFNB1_TARGETS**	92	1.8326064	0.021956088	0.23297375
**BOWIE_RESPONSE_TO_TAMOXIFEN**	18	1.8294877	0.001988072	0.20370016
**FARMER_BREAST_CANCER_CLUSTER_1**	39	1.8857101	0	0.2400618

Table III.I List of 84 differentially expressed gene with higher expression in symptomatic plaques *vs.* asymptomatic plaques. (P < 0.05 and fold-change > 1.4)

Genes / Features	Upregulated In	Feature P	Fold Change	Asymptomatic Mean	Symptomatic Mean
**CSF2RA**	Symptomatic	4.000E-04	1.512	120.497	182.239
**CR1**	Symptomatic	6.000E-04	1.818	342.660	623.027
**BASP1**	Symptomatic	1.400E-03	1.469	1168.274	1715.890
**THEX1**	Symptomatic	1.400E-03	1.443	169.458	244.541
**PQLC3**	Symptomatic	1.600E-03	1.575	219.774	346.091
**CXCL10**	Symptomatic	2.400E-03	1.910	118.407	226.132
**LOC653907**	Symptomatic	2.400E-03	1.730	122.440	211.860
**EML4**	Symptomatic	2.999E-03	1.478	147.411	217.923
**MGAT4A**	Symptomatic	2.999E-03	1.510	371.629	561.196
**C12ORF32**	Symptomatic	3.199E-03	1.401	144.056	201.853
**ARRDC3**	Symptomatic	3.399E-03	1.499	862.086	1291.939
**HS.30495**	Symptomatic	3.399E-03	1.728	117.376	202.800
**CD33**	Symptomatic	4.199E-03	1.415	102.730	145.321
**GCA**	Symptomatic	4.599E-03	1.449	2181.671	3160.166
**FAH**	Symptomatic	5.199E-03	1.540	103.053	158.711
**HS.197143**	Symptomatic	5.399E-03	1.546	349.788	540.744
**MRPS36**	Symptomatic	5.399E-03	1.503	101.357	152.291
**PLCL2**	Symptomatic	5.399E-03	1.412	193.235	272.766
**PLXNC1**	Symptomatic	5.999E-03	1.406	153.754	216.162
**ASGR1**	Symptomatic	6.199E-03	1.739	292.228	508.290
**UBE2E3**	Symptomatic	6.199E-03	1.404	251.298	352.802
**HSPH1**	Symptomatic	8.598E-03	1.491	239.604	357.278
**S100A9**	Symptomatic	8.598E-03	1.588	220.209	349.657
**GNPDA1**	Symptomatic	9.598E-03	1.418	367.808	521.460
**MS4A6A**	Symptomatic	1.020E-02	1.495	693.179	1036.110
**TNFRSF21**	Symptomatic	1.060E-02	1.550	297.303	460.703
**TMEM26**	Symptomatic	1.080E-02	1.622	201.288	326.462
**AP4B1**	Symptomatic	1.120E-02	1.462	108.446	158.591
**P2RY1**	Symptomatic	1.120E-02	1.685	172.176	290.128
**C11ORF75**	Symptomatic	1.160E-02	1.422	668.383	950.480
**IL18BP**	Symptomatic	1.220E-02	1.679	340.228	571.230
**MAP3K8**	Symptomatic	1.280E-02	1.416	121.378	171.892
**IFNGR1**	Symptomatic	1.300E-02	1.460	1062.396	1551.254
**KCNK13**	Symptomatic	1.300E-02	1.642	117.098	192.276
**MAOA**	Symptomatic	1.320E-02	1.449	272.677	394.981
**CXCL9**	Symptomatic	1.360E-02	1.657	99.945	165.651
**CMBL**	Symptomatic	1.380E-02	1.407	105.924	149.087
**CSF3R**	Symptomatic	1.400E-02	1.407	147.717	207.768
**CMKLR1**	Symptomatic	1.560E-02	1.550	198.899	308.334
**PSMD11**	Symptomatic	1.560E-02	1.415	193.652	274.040

**MPEG1**	Symptomatic	1.580E-02	1.480	173.693	256.995
**PFKFB4**	Symptomatic	1.600E-02	1.532	97.721	149.745
**NFS1**	Symptomatic	1.640E-02	1.482	67.173	99.550
**AIF1**	Symptomatic	1.740E-02	1.446	469.147	678.194
**ASCL2**	Symptomatic	1.760E-02	1.410	168.963	238.189
**FGD2**	Symptomatic	1.820E-02	1.404	555.465	779.968
**SIGLEC14**	Symptomatic	1.840E-02	1.602	309.843	496.236
**CA12**	Symptomatic	1.900E-02	1.614	118.924	191.997
**CDC42**	Symptomatic	1.980E-02	1.402	272.390	381.831
**CD163**	Symptomatic	2.000E-02	1.546	357.809	553.190
**PRKACB**	Symptomatic	2.040E-02	1.455	289.136	420.641
**BRSK1**	Symptomatic	2.060E-02	1.487	328.472	488.347
**FOS**	Symptomatic	2.220E-02	1.629	796.454	1297.451
**MRO**	Symptomatic	2.460E-02	1.459	100.491	146.623
**EPSTI1**	Symptomatic	2.480E-02	1.470	432.719	635.992
**PRKCB1**	Symptomatic	2.539E-02	1.524	182.938	278.777
**KCNJ2**	Symptomatic	2.619E-02	1.440	89.238	128.539
**SRP14**	Symptomatic	2.619E-02	1.441	389.523	561.158
**GPR65**	Symptomatic	2.659E-02	1.404	133.485	187.420
**S100A8**	Symptomatic	2.679E-02	1.915	213.599	409.134
**HS.133181**	Symptomatic	2.699E-02	1.457	111.697	162.709
**LOC389386**	Symptomatic	2.719E-02	1.421	180.525	256.451
**SERPINA1**	Symptomatic	2.759E-02	1.499	574.395	860.807
**NICN1**	Symptomatic	2.839E-02	1.402	377.243	529.033
**OLFML2B**	Symptomatic	2.839E-02	1.413	347.968	491.666
**IL2RA**	Symptomatic	3.159E-02	1.768	92.948	164.329
**RPS4Y1**	Symptomatic	3.159E-02	1.482	718.575	1064.642
**GLB1**	Symptomatic	3.339E-02	1.450	385.192	558.446
**CCL8**	Symptomatic	3.359E-02	1.829	508.533	930.105
**ADM**	Symptomatic	3.459E-02	1.912	613.975	1173.670
**SULF2**	Symptomatic	3.479E-02	1.455	104.803	152.449
**GCNT1**	Symptomatic	3.639E-02	1.486	141.400	210.178
**LOC645638**	Symptomatic	3.739E-02	1.475	111.119	163.848
**SAP30**	Symptomatic	3.759E-02	1.410	241.410	340.447
**TGFBI**	Symptomatic	3.759E-02	1.405	809.361	1137.332
**CTSL1**	Symptomatic	3.839E-02	1.591	545.356	867.541
**ZNF185**	Symptomatic	3.839E-02	1.442	138.181	199.224
**LOC100132336**	Symptomatic	3.879E-02	1.496	66.087	98.873
**LOC728401**	Symptomatic	4.179E-02	1.448	105.578	152.845
**LOC730415**	Symptomatic	4.279E-02	1.601	85.080	136.184
**CTSC**	Symptomatic	4.439E-02	1.422	437.284	622.003
**GLUL**	Symptomatic	4.599E-02	1.722	267.502	460.557
**CPVL**	Symptomatic	4.899E-02	1.513	1214.579	1838.211
**PLIN2**	Symptomatic	4.899E-02	1.618	679.753	1099.770

Table III.II List of IPA-derived upstream regulators in macrophages from symptomatic plaques. 31 significant predicted upstream regulators, together with 7 upstream regulators relevant in atherosclerosis that narrowly missed the significant threshold of z-score > 2 or <2.

Upstream Regulator	Molecular Type	Predicted Activation State	Activation z-score	Overlap P-val
**IL1B**	cytokine	Activated	3.138	5.35E-06
**TNF**	cytokine	Activated	3.046	3.52E-11
**IL1**	group	Activated	2.922	7.88E-07
**PDGF BB**	complex	Activated	2.903	5.05E-06
**IL1A**	cytokine	Activated	2.733	1.68E-07
**RELA**	transcription regulator	Activated	2.587	6.32E-04
**CEBPA**	transcription regulator	Activated	2.451	9.79E-09
**NFKB1**	transcription regulator	Activated	2.416	4.00E-06
**PI3K (family)**	group	Activated	2.401	4.64E-07
**NFkB (complex)**	complex	Activated	2.264	3.50E-06
**E. coli B5 lipopolysaccharide**	chemical	Activated	2.219	3.32E-03
**NFKBIA**	transcription regulator	Activated	2.215	5.47E-05
**AKT1**	kinase	Activated	2.201	2.55E-03
**ERK**	group	Activated	2.193	2.11E-03
**Ins1**	other	Activated	2.173	1.61E-03
**IRF7**	transcription regulator	Activated	2.170	4.93E-04
**AGT**	growth factor	Activated	2.169	8.82E-06
**PPARA**	nuclear receptor	Activated	2.169	3.78E-05
**ERK1/2**	group	Activated	2.156	1.08E-04
**HIF1A**	transcription regulator	Activated	2.128	1.68E-06
**Forskolin**	chemical toxicant	Activated	2.099	1.15E-03
**Peptidoglycan**	chemical	Activated	2.060	3.16E-06
**EHF**	transcription regulator	Activated	2.000	2.81E-04
**STAT1**	transcriptional regulator	Activated	2.000	4.59E-03
**ARNT2**	transcription regulator	Activated	2.000	9.31E-03
**RAF1**	kinase	Activated	2.000	9.94E-03
**TGM2**	enzyme	Activated	2.000	1.26E-02
**FAS**	transmembrane receptor	-	1.981	6.63E-04
**MAPK14**	kinase	-	1.981	3.94E-03

**NOS2**	enzyme	-	1.974	6.67E-03
**STAT5B**	transcriptional regulator	-	1.964	4.21E-03
**IL18**	cytokine	-	1.952	2.15E-03
**MYD88**	other	-	1.935	5.13E-06
**SOCS3**	phosphatase	-	-1.945	2.30E-04
**ERBB3**	kinase	Inhibited	-2.000	3.25E-04
**IL13**	cytokine	Inhibited	-2.065	1.69E-02
**INSIG1**	other	Inhibited	-2.236	5.51E-05
**Nitrofurantoin**	chemical drug	Inhibited	-2.236	5.35E-04

Table III.III List of IPA-derived downstream effects and predicted cellular functions based on gene expression changes found in macrophages from symptomatic plaques. 17 significantly increased cellular functions was listed, together with 8 other relevant increased cellular functions that narrowly missed z-score threshold.

Cellular Function	Predicted Activation State	Activation z-score	P-val
**Homing of cells**	Increased	2.977	8.10E-08
**Chemotaxis of cells**	Increased	2.830	2.20E-07
**Chemotaxis of phagocytes**	Increased	2.646	4.80E-09
**Chemotaxis of myeloid cells**	Increased	2.639	4.06E-08
**Synthesis of nitric oxide**	Increased	2.516	6.13E-04
**Proliferation of vascular endothelial cells**	Increased	2.442	6.69E-05
**Chemotaxis of granulocytes**	Increased	2.423	5.72E-07
**Engulfment of cells**	Increased	2.400	7.11E-05
**Lymphocyte migration**	Increased	2.389	9.83E-05
**Adhesion of blood cells**	Increased	2.249	4.21E-03
**Chemotaxis of neutrophils**	Increased	2.204	1.68E-06
**Mitogenesis**	Increased	2.190	1.55E-04
**Cell death of tumor cells**	Increased	2.186	3.69E-03
**Phagocytosis of cells**	Increased	2.124	1.14E-05
**Development of epithelial tissue**	Increased	2.053	2.90E-05
**Proliferation of endothelial cells**	Increased	2.045	6.78E-07

**Quantity of metal**	Increased	2.029	1.98E-09
**Quantity of Ca2+**	-	1.979	5.48E-09
**Migration of phagocytes**	-	1.970	8.39E-05
**Release of nitric oxide**	-	1.969	5.84E-04
**Recruitment of T lymphocytes**	-	1.969	2.99E-04
**Concentration of Ca2+**	-	1.967	2.88E-03
**Chemotaxis of monocytes**	-	1.964	1.06E-03
**Migration of mononuclear leukocytes**	-	1.962	3.18E-05
**Chemotaxis of mononuclear leukocytes**	-	1.947	2.23E-03

Table III.IV List of 60 differentially expressed genes with higher expression in macrophages from plaques with Lipid Area >25%. (P < 0.05 and fold-change > 1.5)

Description	Upregulated In	Feature P	Fold Change	Low_lipid Mean	High_lipid Mean
**RSAD2**	high_lipid	3.999E-04	2.005	82.316	165.046
**IFIT1**	high_lipid	9.998E-04	2.117	328.160	694.561
**CXCL10**	high_lipid	1.200E-03	2.104	107.834	226.911
**MX1**	high_lipid	1.200E-03	1.503	1497.968	2252.140
**CXCL9**	high_lipid	1.400E-03	1.832	91.664	167.959
**EPSTI1**	high_lipid	1.400E-03	1.756	381.078	669.154
**MCM10**	high_lipid	1.400E-03	1.510	104.286	157.432
**IFNGR1**	high_lipid	1.600E-03	1.559	1003.958	1565.250
**STAT1**	high_lipid	1.600E-03	1.629	389.897	635.290
**IFIT3**	high_lipid	2.200E-03	1.940	309.199	599.789
**KCNK13**	high_lipid	2.400E-03	1.791	108.381	194.158
**C20ORF24**	high_lipid	2.799E-03	1.577	155.316	244.943
**JAKMIP2**	high_lipid	2.799E-03	1.514	71.072	107.575
**RNF144B**	high_lipid	3.799E-03	1.508	193.214	291.280
**OAS2**	high_lipid	3.999E-03	1.645	153.865	253.092
**ISG15**	high_lipid	4.599E-03	1.763	192.535	339.483
**P2RY1**	high_lipid	5.199E-03	1.754	163.955	287.626
**GLB1**	high_lipid	5.999E-03	1.611	355.414	572.473
**IFIT2**	high_lipid	6.599E-03	1.717	205.605	352.979
**IL18BP**	high_lipid	6.999E-03	1.739	325.084	565.374
**PSMA6**	high_lipid	7.199E-03	1.568	216.736	339.863

**C6ORF173**	high_lipid	7.798E-03	1.533	114.599	175.660
**SNX10**	high_lipid	7.998E-03	1.583	282.446	447.150
**IFI6**	high_lipid	8.998E-03	1.610	161.936	260.666
**SIGLEC14**	high_lipid	1.060E-02	1.663	296.363	492.770
**TMEM26**	high_lipid	1.100E-02	1.659	194.210	322.161
**HS.30495**	high_lipid	1.120E-02	1.646	118.070	194.340
**PLIN2**	high_lipid	1.140E-02	1.809	619.809	1121.531
**IFIT3**	high_lipid	1.340E-02	1.504	98.016	147.396
**C19ORF59**	high_lipid	1.460E-02	1.995	83.496	166.586
**ADAMDEC1**	high_lipid	1.480E-02	2.651	160.699	425.945
**DUSP1**	high_lipid	1.480E-02	1.514	1246.441	1887.526
**CCL4L1**	high_lipid	1.620E-02	1.583	298.659	472.747
**KLHDC8B**	high_lipid	1.800E-02	1.501	733.995	1101.571
**CTSL1**	high_lipid	2.120E-02	1.683	515.702	867.906
**CR1**	high_lipid	2.160E-02	1.537	370.542	569.657
**SMPDL3A**	high_lipid	2.220E-02	1.655	238.586	394.820
**ACSL3**	high_lipid	2.320E-02	1.574	216.806	341.318
**PRKCB1**	high_lipid	2.320E-02	1.529	179.096	273.906
**LOC100132336**	high_lipid	2.460E-02	1.506	64.645	97.334
**S100A9**	high_lipid	2.559E-02	1.504	222.872	335.226
**IFI44L**	high_lipid	2.599E-02	1.655	136.862	226.446
**PGD**	high_lipid	2.699E-02	1.703	261.803	445.757
**ACOT11**	high_lipid	2.799E-02	1.685	428.070	721.099
**LOC645313**	high_lipid	2.799E-02	1.663	163.342	271.665
**SCD**	high_lipid	3.059E-02	1.625	1455.404	2364.906
**IL4I1**	high_lipid	3.279E-02	1.514	88.019	133.237
**FOS**	high_lipid	3.299E-02	1.574	795.699	1252.661
**GLUL**	high_lipid	3.379E-02	1.768	256.710	453.799
**ASGR1**	high_lipid	3.699E-02	1.538	307.641	473.235
**MGST1**	high_lipid	3.819E-02	1.691	78.855	133.334
**S100A8**	high_lipid	3.819E-02	1.849	212.323	392.634
**UBD**	high_lipid	3.819E-02	1.576	97.157	153.079
**IL1RN**	high_lipid	3.879E-02	1.887	97.084	183.213
**FABP5**	high_lipid	4.179E-02	1.507	1277.206	1925.358
**PLA2G7**	high_lipid	4.179E-02	1.504	879.091	1322.285
**IDH1**	high_lipid	4.219E-02	1.569	350.095	549.399
**IFI27**	high_lipid	4.519E-02	1.567	984.630	1543.398
**AADACL1**	high_lipid	4.539E-02	1.521	223.357	339.820
**BCL2A1**	high_lipid	4.819E-02	1.617	841.604	1360.944

Table III.V List of 17 significant IPA-derived upstream regulators in macrophages from plaque with large lipid core (>25% Lipid Area on T2 map).

Upstream Regulator	Molecular Type	Predicted Activation State	Activation z-score	Overlap P-val
**IFNG**	cytokine	Activated	3.917	9.56E-12
**IFNL1**	cytokine	Activated	3.552	7.73E-18
**IFNA2**	cytokine	Activated	3.439	2.66E-14
**TGM2**	enzyme	Activated	3.162	1.54E-07
**PRL**	cytokine	Activated	3.092	8.33E-12
**Interferon alpha**	group	Activated	2.374	2.38E-07
**IFNA1/IFNA13**	cytokine	Activated	2.364	6.39E-13
**PAF1**	other	Activated	2.219	5.07E-06
**TNF**	cytokine	Activated	2.204	1.57E-08
**EIF2AK2**	kinase	Activated	2.2	1.35E-05
**P38 MAPK**	group	Activated	2.194	8.73E-04
**IRF3**	transcription regulator	Activated	2.189	6.70E-07
**IgG**	complex	Inhibited	-2	3.76E-03
**SOCS3**	phosphatase	Inhibited	-2.236	8.51E-09
**MAPK1**	kinase	Inhibited	-2.345	2.61E-07
**SOCS1**	other	Inhibited	-2.39	1.23E-08
**IL1RN**	cytokine	Inhibited	-2.985	1.16E-10

Table III.VI List of 7 significant IPA-derived downstream cellular functions in macrophages from plaque with large lipid core (>25% Lipid Area on T2 map).

Cellular Function	Predicted Activation State	Activation z-score	P-val
**Proliferation of cells**	Increased	2.977	2.05E-07
**Immune response of cells**	Increased	2.830	2.50E-03
**Relapsing-remitting multiple sclerosis**	Increased	2.646	2.81E-05
**Chemotaxis of phagocytes**	Increased	2.639	6.78E-06
**Inflammatory response**	Increased	2.516	3.38E-04
**Chemotaxis of myeloid cells**	Increased	2.442	9.61E-06
**Chemotaxis of granulocytes**	Increased	2.423	4.85E-06

Table IV List of 314 human SNPs with susceptibility association to coronary artery disease (derived from the interim UK Biobank (UKBB) report) and ischaemic stroke (METASTROKE) used in MAGENTA analysis

rsid	Dataset	rsid	Dataset	rsid	Dataset
rs2843152	Nelson et al	rs13200993	Nelson et al	rs11057401	Nelson et al
rs35465346	Nelson et al	rs3130683	Nelson et al	rs11057830	Nelson et al
rs7538207	Nelson et al	rs4472337	Nelson et al	rs1924981	Nelson et al
rs11811081	Nelson et al	rs56015508	Nelson et al	rs9591012	Nelson et al
rs12733730	Nelson et al	rs1214752	Nelson et al	rs73468973	Nelson et al
rs11485595	Nelson et al	rs6905288	Nelson et al	rs75535189	Nelson et al
rs34232196	Nelson et al	rs1330633	Nelson et al	rs11617955	Nelson et al
rs11591147	Nelson et al	rs194937	Nelson et al	rs7139492	Nelson et al
rs17111652	Nelson et al	rs11153071	Nelson et al	rs4773141	Nelson et al
rs6665249	Nelson et al	rs9398803	Nelson et al	rs11838776	Nelson et al
rs56170783	Nelson et al	rs12202017	Nelson et al	rs35166119	Nelson et al
rs112470402	Nelson et al	rs9493752	Nelson et al	rs61969072	Nelson et al
rs2149821	Nelson et al	rs2492304	Nelson et al	rs17102313	Nelson et al
rs10890013	Nelson et al	rs2153219	Nelson et al	rs12891473	Nelson et al
rs113832197	Nelson et al	rs139311851	Nelson et al	rs4506804	Nelson et al
rs7528419	Nelson et al	rs202220802	Nelson et al	rs3832966	Nelson et al
rs11552449	Nelson et al	rs202097157	Nelson et al	rs112635299	Nelson et al
rs10305649	Nelson et al	rs147555597	Nelson et al	rs10139550	Nelson et al
rs11810571	Nelson et al	rs41272114	Nelson et al	rs113025579	Nelson et al
rs6689306	Nelson et al	rs10455872	Nelson et al	rs147580454	Nelson et al
rs2789422	Nelson et al	rs79018195	Nelson et al	rs72743461	Nelson et al
rs6413828	Nelson et al	rs41269888	Nelson et al	rs7177201	Nelson et al
rs183692864	Nelson et al	rs186696265	Nelson et al	rs7164479	Nelson et al
rs2820315	Nelson et al	rs6956990	Nelson et al	rs2083460	Nelson et al
rs67180937	Nelson et al	rs12535339	Nelson et al	rs2071382	Nelson et al
rs3755549	Nelson et al	rs11509880	Nelson et al	rs17581137	Nelson et al
rs2709437	Nelson et al	rs2107595	Nelson et al	rs116082507	Nelson et al
rs6727557	Nelson et al	rs55889159	Nelson et al	rs7185993	Nelson et al
rs16986953	Nelson et al	rs78850423	Nelson et al	rs247616	Nelson et al
rs585967	Nelson et al	rs2971672	Nelson et al	rs35259348	Nelson et al
rs58560619	Nelson et al	rs1088868	Nelson et al	rs9929108	Nelson et al
rs71737208	Nelson et al	rs148475353	Nelson et al	rs7500448	Nelson et al
rs4299376	Nelson et al	rs35146811	Nelson et al	rs1968266	Nelson et al
rs4076834	Nelson et al	rs112370447	Nelson et al	rs7205284	Nelson et al
rs139591697	Nelson et al	rs2024233	Nelson et al	rs117592425	Nelson et al
rs72375964	Nelson et al	rs11556924	Nelson et al	rs113348108	Nelson et al
rs7568458	Nelson et al	rs2286198	Nelson et al	rs8068571	Nelson et al
rs149366039	Nelson et al	rs3918226	Nelson et al	rs9897596	Nelson et al
rs7578433	Nelson et al	rs2083636	Nelson et al	rs13723	Nelson et al
rs79716828	Nelson et al	rs28597716	Nelson et al	rs148720362	Nelson et al

rs6761276	Nelson et al	rs367948	Nelson et al	rs1122326	Nelson et al
rs201837187	Nelson et al	rs16885577	Nelson et al	rs8068844	Nelson et al
rs7570006	Nelson et al	rs10109493	Nelson et al	rs4643373	Nelson et al
rs17678683	Nelson et al	rs72658939	Nelson et al	rs62076439	Nelson et al
rs35500812	Nelson et al	rs77211063	Nelson et al	rs8068952	Nelson et al
rs12619842	Nelson et al	rs10955380	Nelson et al	rs6504218	Nelson et al
rs62172372	Nelson et al	rs2954029	Nelson et al	rs11077501	Nelson et al
rs114123510	Nelson et al	rs117938894	Nelson et al	rs75589791	Nelson et al
rs2011559	Nelson et al	rs75824083	Nelson et al	rs35489971	Nelson et al
rs1250229	Nelson et al	rs58594043	Nelson et al	rs11654510	Nelson et al
rs2161967	Nelson et al	rs34914400	Nelson et al	rs7211674	Nelson et al
rs2972146	Nelson et al	rs199997514	Nelson et al	rs9951447	Nelson et al
rs10168194	Nelson et al	rs2891168	Nelson et al	rs178002	Nelson et al
rs13003675	Nelson et al	rs1333050	Nelson et al	rs12922	Nelson et al
rs10929113	Nelson et al	rs4149311	Nelson et al	rs833509	Nelson et al
rs143803699	Nelson et al	rs1967604	Nelson et al	rs948937	Nelson et al
rs748431	Nelson et al	rs111245230	Nelson et al	rs35614134	Nelson et al
rs3821396	Nelson et al	rs781622	Nelson et al	rs116843064	Nelson et al
rs7623687	Nelson et al	rs77275410	Nelson et al	rs111397563	Nelson et al
rs77622129	Nelson et al	rs10818583	Nelson et al	rs6511720	Nelson et al
rs62253653	Nelson et al	rs507666	Nelson et al	rs2738448	Nelson et al
rs62265630	Nelson et al	rs11257613	Nelson et al	rs167479	Nelson et al
rs71331765	Nelson et al	rs7094201	Nelson et al	rs73015715	Nelson et al
rs6787409	Nelson et al	rs1887318	Nelson et al	rs78030362	Nelson et al
rs139016349	Nelson et al	rs1870634	Nelson et al	rs10423964	Nelson et al
rs4632520	Nelson et al	rs1657345	Nelson et al	rs10417115	Nelson et al
rs12493885	Nelson et al	rs17726488	Nelson et al	rs34322801	Nelson et al
rs10513507	Nelson et al	rs4691	Nelson et al	rs73045269	Nelson et al
rs34229028	Nelson et al	rs7098414	Nelson et al	rs8108632	Nelson et al
rs9869263	Nelson et al	rs2246942	Nelson et al	rs4760	Nelson et al
rs113148244	Nelson et al	rs59898454	Nelson et al	rs7412	Nelson et al
rs16994919	Nelson et al	rs11191416	Nelson et al	rs56131196	Nelson et al
rs200686624	Nelson et al	rs12252333	Nelson et al	rs1964272	Nelson et al
rs2616407	Nelson et al	rs2257129	Nelson et al	rs425105	Nelson et al
rs13134452	Nelson et al	rs2281674	Nelson et al	rs13734	Nelson et al
rs72627509	Nelson et al	rs28596486	Nelson et al	rs59909520	Nelson et al
rs10857147	Nelson et al	rs56210063	Nelson et al	rs117113213	Nelson et al
rs36002015	Nelson et al	rs10840293	Nelson et al	rs6129767	Nelson et al
rs138495951	Nelson et al	rs3993105	Nelson et al	rs56313611	Nelson et al
rs7678555	Nelson et al	rs11462682	Nelson et al	rs259983	Nelson et al
rs144059514	Nelson et al	rs146039567	Nelson et al	rs3813452	Nelson et al
rs13109172	Nelson et al	rs2306029	Nelson et al	rs2832275	Nelson et al
rs4593108	Nelson et al	rs2727020	Nelson et al	rs75187018	Nelson et al
rs6841581	Nelson et al	rs12146487	Nelson et al	rs28451064	Nelson et al
rs7435973	Nelson et al	rs12801636	Nelson et al	rs743339	Nelson et al
rs3796587	Nelson et al	rs571353	Nelson et al	rs117696200	Nelson et al
rs869396	Nelson et al	rs634552	Nelson et al	rs2836621	Nelson et al
rs11728590	Nelson et al	rs3133293	Nelson et al	rs35219138	Nelson et al

rs71600236	Nelson et al	rs17712139	Nelson et al	rs9604969	Nelson et al
rs112941079	Nelson et al	rs2212437	Nelson et al	rs71313931	Nelson et al
rs5868014	Nelson et al	rs2839812	Nelson et al	rs11287675	Nelson et al
rs111777100	Nelson et al	rs567040	Nelson et al	rs12485143	Nelson et al
rs288187	Nelson et al	rs964184	Nelson et al	rs180803	Nelson et al
rs1800449	Nelson et al	rs3782774	Nelson et al	rs468224	Nelson et al
rs1500187	Nelson et al	rs72447384	Nelson et al	rs10179686	METASTROKE
rs6883598	Nelson et al	rs3861086	Nelson et al	rs6792835	METASTROKE
rs251023	Nelson et al	rs11170820	Nelson et al	rs17167021	METASTROKE
rs11955380	Nelson et al	rs56245751	Nelson et al	rs632728	METASTROKE
rs3776307	Nelson et al	rs11172113	Nelson et al	rs7791394	METASTROKE
rs6860540	Nelson et al	rs2229357	Nelson et al	rs1333047	METASTROKE
rs9501744	Nelson et al	rs6538176	Nelson et al	rs7937106	METASTROKE
rs421329	Nelson et al	rs11115214	Nelson et al	rs4304924	METASTROKE
rs742115	Nelson et al	rs2681472	Nelson et al	rs1005224	METASTROKE
rs6458138	Nelson et al	rs10774625	Nelson et al	rs1465330	METASTROKE
rs9349379	Nelson et al	rs2244608	Nelson et al		

Table V List of enriched pathways with a nominal P-value < 0.05 in the additional MAGENTA analysis using new SNPs list from Circ Res 2018 Feb 2; 122(3): 433-443 paper.

Database	GeneSet	NOMINAL GSEA P-VAL
GOTERM	protein heterodimerization activity	1.00E-03
Panther	Wnt_signaling_pathway	1.90E-03
Ingenuity	FXR.RXR.Activation	2.50E-03
GOTERM	wound healing	3.10E-03
REACTOME	METABOLISM_OF_LIPIDS_AND_LIPOPROTEINS	5.70E-03
GOTERM	cell surface	1.08E-02
GOTERM	heme binding	1.11E-02
GOTERM	cholesterol catabolic process	1.29E-02
GOTERM	caveola	1.39E-02
GOTERM	blood circulation	1.43E-02
GOTERM	ubiquitin-dependent protein catabolic process	1.45E-02
GOTERM	trans-Golgi network	1.46E-02
GOTERM	cholesterol homeostasis	2.33E-02
GOTERM	cholesterol metabolic process	2.41E-02
GOTERM	lipid transport	2.42E-02
GSEA	REACTOME_LIPID_DIGESTION_MOBILIZATION_AND_TRANSPORT	2.72E-02
GOTERM	regulation of transcription	2.75E-02
GOTERM	endoplasmic reticulum membrane	2.82E-02
GOTERM	neuronal cell body	3.47E-02
GOTERM	response to drug	4.03E-02
GOTERM	lipid metabolic process	4.20E-02
PANTHER_BIOLOGICAL_PROCESS	Cell_adhesion-mediated_signaling	4.32E-02
GOTERM	endosome membrane	4.68E-02
GOTERM	low-density lipoprotein particle	4.69E-02
GOTERM	aging	4.76E-02
GOTERM	cholesterol efflux	4.76E-02
GOTERM	microsome	4.82E-02
REACTOME	STEROID_METABOLISM	4.98E-02
