# Supplementary material for: Differential Gene Expression in Macrophages From Human Atherosclerotic Plaques Shows Convergence on Pathways Implicated by Genome-Wide Association Study Risk Variants
Source: Arterioscler Thromb Vasc Biol. 2018 Sep 6;38(11):2718–30. doi: 10.1161/ATVBAHA.118.311209 (PMC6217969; doi:10.1161/ATVBAHA.118.311209)
Supplement: Supplementary file 4 [file atv-38-2718-s004.pdf]

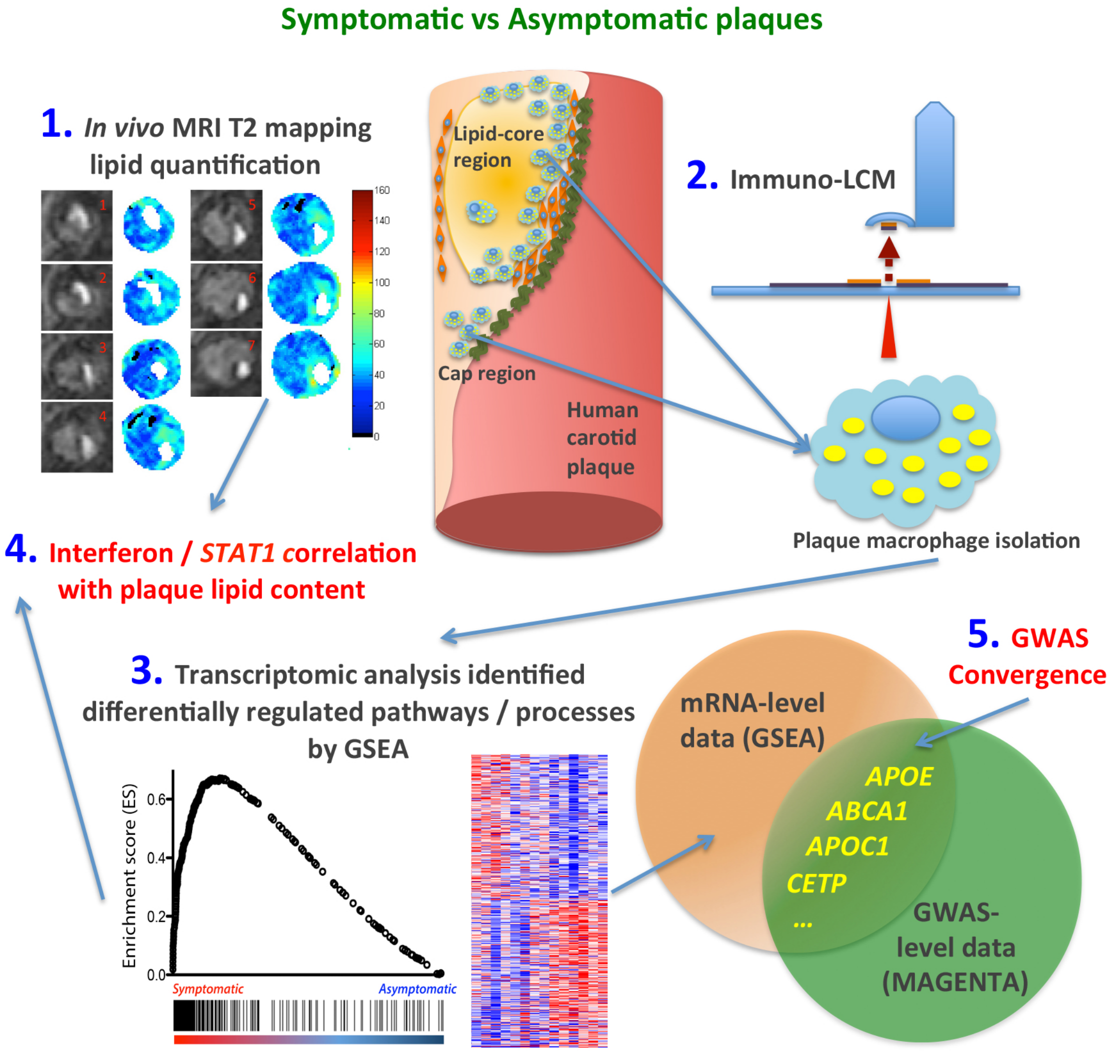

Symptomatic vs Asymptomatic plaques
1. In vivo MRI T2 mapping lipid quantification
Lipid-core region
Cap region
Human carotid plaque
2. Immuno-LCM
Plaque macrophage isolation
4. Interferon / STAT1 correlation with plaque lipid content
3. Transcriptomic analysis identified differentially regulated pathways / processes by GSEA
Enrichment score (ES)
Symptomatic
Asymptomatic
5. GWAS Convergence
mRNA-level data (GSEA)
APOE
ABCA1
APOC1
CETP
...
GWAS-level data (MAGENTA)
